# Unpalatable plants induce a species-specific associational effect on neighboring communities

**DOI:** 10.1038/s41598-021-93698-4

**Published:** 2021-07-13

**Authors:** Mohammad Bagher Erfanian, Farshid Memariani, Zohreh Atashgahi, Mansour Mesdaghi, Maliheh Saeedi, Mojtaba Darrudi, Maliheh Hamedian, Saeede Hosseini, Hamid Ejtehadi

**Affiliations:** 1 Quantitative Plant Ecology and Biodiversity Research Laboratory, Department of Biology, Faculty of Science, Ferdowsi University of Mashhad, PO BOX 9177948974, Mashhad, Iran; 2 Herbarium FUMH, Department of Botany, Research Center for Plant Sciences, Ferdowsi University of Mashhad, Mashhad, Iran; 3 Department of Range and Watershed Management, Faculty of Natural Resources and Environment, Ferdowsi University of Mashhad, Mashhad, Iran

**Keywords:** Biodiversity, Community ecology, Grassland ecology

## Abstract

In grazing conditions, unpalatable species may induce either associational defense or neighbor contrast susceptibility in neighboring communities. Using surveys from eight grasslands, we tested whether various unpalatable species have the same impacts on neighboring communities in response to grazing. The studied unpalatable species were: *Phlomis cancellata* (an unpalatable nonpoisonous plant), *Euphorbia boissieriana*, *E.* *microsciadia* (poisonous plants), and *Seseli transcaucasicum* (a highly poisonous plant). Our results showed that, in the ungrazed grasslands, communities containing *P*. *cancellata* had lower biodiversity than communities without it. In the moderately- and heavily grazed grasslands, *P*. *cancellata* induced associational defense in the neighboring communities. In heavily grazed grasslands, both *Euphorbia* species promoted neighbor contrast susceptibility in the neighboring communities. Similarly, *S*. *transcaucasicum* in a heavily grazed grassland, induced neighbor contrast susceptibility. Different responses of plant community vulnerability among the studied unpalatable plants might be due to herbivore different foraging decisions. Accordingly, grazers selectively choose from other patches when facing *P*. *cancellata* and other plant individuals when there is a poisonous plant in a patch. Our results suggested that grazing intensity may not substantially affect the foraging decisions of sheep and goats in response to unpalatable species. We recommend monitoring the abundance of poisonous species to maintain the sustainable use of grasslands.

## Introduction

Foraging decisions of large herbivores in order to select species with high nutrients and energy affect plant communities^1^. Plant species adapt to grazing by using morphological and chemical defensive traits^2^. Defensive traits of neighboring species modify the degree of protection of a focal species against large herbivores^3–5^. Thus, whether species gain defense or become more susceptible to grazing also depends on its neighbors^5,6^.

In grazing conditions, plant species in a community can promote four types of associational effects: (1) Associational defense, plants can reduce herbivore damage by growing closely to unpalatable neighbors. (2) Associational susceptibility, plants experience increased damage by growing together with more palatable species. (3) Neighbor contrast defense, plants will be less chosen by neighboring with more palatable species. (4) Neighbour contrast susceptibility, plants will be more susceptible to herbivory by neighboring with a less palatable species^1^. Therefore, unpalatable species can induce either associational defense or neighbor contrast susceptibility. For livestock grazing, associational defense is more likely to happen than neighbor contrast susceptibility^5^.

Previous studies on evaluating the effects of unpalatable species on neighboring communities have tended to focus on plants that use structural defensive traits (e.g., Refs.^7–9^). Consequently, species using chemical defensive traits have rarely been studied. Moreover, there is inconsistency among the reported findings on the impacts of poisonous species on neighboring communities. For example, both negative (e.g., Refs.^10,11^) and positive impacts—e.g., Refs.^4,10,12^—have been reported.

Most of the research have been limited to study a single focal species, and the effects of unpalatable species at the community level received lesser attention^13,14^. Although phylogenetic diversity can be altered by different plant–plant interactions^15^, no studies have compared this measure between communities containing or without a chemically unpalatable or poisonous species to the best knowledge of the authors. Generally, in the literature on associational effects of unpalatable species, effects of grazing intensity have been neglected. Nevertheless, it is well-established that interaction between plants is likely to be changed with different grazing intensities^6,16^.

Here, we use eight datasets that were collected from ungrazed, moderately-, and heavily grazed sites in northeastern Iran. We evaluated the species and phylogenetic diversities as well as the species composition of plant communities containing and without four unpalatable plants: a nonpoisonous chemically unpalatable, two poisonous, and a highly poisonous species. We hypothesized that chemically unpalatable plants—with different toxicity levels—do not show the same associational effect. The aim of this study was to quantify the impacts of these species on biodiversity and species composition of neighboring communities. We compare these communities with communities without unpalatable species in different levels of large herbivore grazing.

## Materials and methods

### Study areas and datasets

We used data from eight surveys (Table 1) that were sampled from various landscapes in the mountainous ranges of northeastern Iran. Herbaceous plants were the dominant species in the selected sites. Land use change and grazing by sheep and goats were the primary disturbances in the study sites^17–22^. Two sites were partially-managed: the Kelilagh no-hunting zone (hereafter is called the Kelilagh) and the Heydari Wildlife Refuge (hereafter is called the Heydari). The other sites were neither protected nor restored. Information about all sites is presented in Table 1. The selected sites represent moderately- (i.e., the Fereizi and Heydari sites), heavily grazed (i.e., the Darrud, Arabchah, Zharf, and Boghmech sites), and ungrazed areas (i.e., the Najafi and Kelilagh sites).

**Table 1:** Eight datasets were used in this study.

Study areas									
Site name		Arabchah	Boghmech	Darrud	Fereizi	Heydari	Kelilagh	Najafi	Zharf
Coordinates	N	36.992	36.838	36.156	36.482	36.730	36.316	36.273	35.500
	E	59.391	59.245	59.150	58.974	58.600	60.000	59.494	59.500
Climate		Semi-arid	Semi-arid	Semi-arid	Semi-arid	Semi-arid	Semi-arid	Semi-arid	Semi-arid
Grazing cond		Heavy	Heavy	Heavy	Moderate	Moderate	Ungrazed	Ungrazed	Heavy
Elevation (m a.s.l.)		2500–2900	1600–2300	1900–2500	1300–1600	2000–2500	1200–1500	1300–1600	1200–1500
Sampling date		2016	2016	2014	2006	2016	2014	2014	2014
Number of analyzable plots		38	18	25	51	20	29	16	34
Sampling unit area		1 m × 1 m	1 m × 1 m	1 m × 1 m	3 m × 3 m	1 m × 1 m	1 m × 1 m	1 m × 1 m	1 m × 1 m
Studied species		Setr	Eumi	Phca; Eubo	Phca	Phca	Phca	Phca	Phca

They were sampled from different mountainous rangelands of northeastern Iran.

Abbreviations: Phca, *Phlomis cancellata*; Eumi, *Euphorbia microsciadia*; Eubo, *E. boissieriana*; Setr, *Seseli transcaucasicum*; Grazing Cond., grazing condition.

### Selected species

We considered the literature data as well as field observations to select unpalatable plants. Four species, ranging from nonpoisonous unpalatable to a highly poisonous plant, were selected:

(A) *Phlomis cancellata* (Lamiaceae) is a fragrant, chemically unpalatable species. This plant is used in the traditional medicine of Iran^19,23^. *Phlomis cancellata* is a dominant species in the grazed areas of northeastern Iran^19^. This species is not poisonous and has a hemicryptophyte life form^24^.
(B) *Euphorbia boissieriana* and *E*. *microsciadia* (Euphorbiaceae) are two poisonous species with a similar physiognomy (i.e., hemicryptophyte)^24^. These plants remain intact after grazing. If they are accidentally ingested, these species will cause severe harm to livestock^25^.
(C) *Seseli transcaucasicum* (Apiaceae) remains intact after grazing. This plant is considered a highly poisonous species that is lethal if ingested by livestock. *S*. *transcaucasicum* has a chamaephyte life form^26^.

### Data preparation

In each dataset, random plots containing > 10% canopy cover of unpalatable species were selected. These plots hereafter are called Contain Focal Species (CFSs). Given the size of the studied species, we estimated that there was at least one full-grown individual in plots that the focal species has a canopy cover of > 10%. Additionally, the nearest plots with no unpalatable species were selected. These plots hereafter are called Without Focal Species (WFSs). Environmental factors (e.g., elevation, aspect, slope degree) were evaluated to be the same to eliminate undesired sources of variability between CFSs and WFSs. If environmental conditions were not the same for a plot pair, this pair was excluded from further analyses. Finally, data from 180 sampling units of 1 m × 1 m and 51 sampling units of 3 m × 3 m area were analyzed. The plots of the Fereizi had a 9 m^2^ area, and those of the other sites had a 1 m^2^ area. We only compared CFSs and WFSs of each site, and no comparisons were made among the sites. We considered the vascular plant species and their canopy cover in WFSs and CFSs.

For *P*. *cancellata*, data from six sites were analyzable. For *Euphorbia* species, two sites and for *S*. *transcaucasicum,* one site had analyzable data. A data was considered analyzable if it had at least five CFSs-WFSs pairs. The data limitation was due to a limited distribution range of unpalatable species, e.g., *S*. *transcaucasicum* has only been recorded in the Arabchah. Further, *Euphorbia* species were rarely recorded from the ungrazed and moderately grazed sites. The data is presented in Supplementary file.

### Data analysis

#### Species diversity

We compared the species diversity of the CFSs and WFSs. We used Hill numbers^27^. We calculated the species richness (q = 0 in the Hill numbers) and the reciprocal of the Gini-Simpson index (q = 2 in the Hill numbers). These indices evaluate the species diversity of a community at the level of rare and dominant species^27^, respectively. A coverage-based rarefaction and extrapolation method was employed for biodiversity calculations. This method was used to eliminate the effects of unequal sampling effort (e.g., an unequal number of samples) on our inferences^28^. We calculated the base coverage of each dataset. We used the method of Chao and Jost^28^ to calculate this value. Then, Species diversity and phylogenetic diversity indices were estimated at the base coverage. A 95% confidence interval (CI) for each index was estimated by using a bootstrap method. All species diversity calculations were conducted using the iNEXT package^29^. For each site, the results were reported as the percentage change in species diversity in CFSs relative to the species diversity of the WFSs.

##### Phylogenetic diversity

We used an angiosperm phylogeny of all vascular species that were collected in the plots. The non-angiosperms were excluded to prevent adding long branches that lead to the outliers generating in the phylogenetic diversity calculations^30^. The phylogenetic trees were created using the V.Phylomaker package^31^. We used the Phylogenetic Hill diversity indices to compare CFSs and WFSs in each site. The phylogenetic diversity at the level of rare (q = 0) and dominant (q = 2) species was calculated. We used a coverage-based rarefaction and extrapolation approach. A 95% CI was also estimated for each index. The iNEXT‐PD package was used for phylogenetic diversity calculations^29,32,33^. The results were reported as the percentage change in phylogenetic diversity of CFSs relative to the phylogenetic diversity of the WFSs.

#### Species composition

We used a transformation-based Principal Component Analysis (tb-PCA) to visualize the variation in species composition of CFSs and WFSs in each site. First, we applied the Hellinger transformation on the species data to eliminate the effects of double zeros on the next analyses^34^. This transformation was performed by using the vegan package^35^. Then, a PCA was conducted on the transformed data by using the *rda* function in the vegan package. We conducted an analysis of similarity (ANOSIM), number of permutations = 999, to test whether the species composition of CFSs and WFSs was significantly different. We used the *anosim* function in the vegan package for this analysis. All analyses were performed in R version 3.6^36^.

## Results

### *Phlomis cancellata*

**Figure 1:**
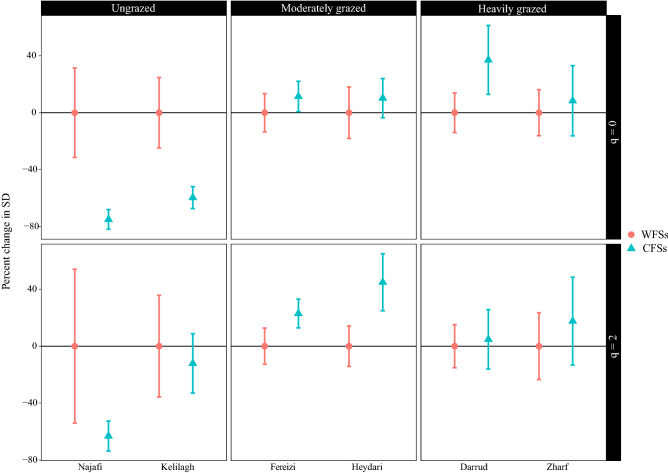
The coverage-based comparison of Hill species diversity of the plant communities containing (CFSs) and without (WFSs) *Phlomis cancellata* in different grazing conditions. The species richness (q = 0) and the reciprocal of the Gini-Simpson index (q = 2) were reported in this study. SD = species diversity. The results are reported as the percentage change of SD in CFSs comparing to that of WFSs.

In the Najafi—an ungrazed site, CFSs showed a 75% lower species richness (q = 0) and 63% lower the reciprocal of the Gini-Simpson index (q = 2) compared to WFSs. The decrease in the species richness was significant in this site (Fig. 1). In the other ungrazed site—the Kelilagh, CFSs had a 60% lower species richness and 13% lower the reciprocal of the Gini-Simpson index than WFSs. In the Kelilagh, the species richness decrease was significant (Fig. 1). On average in the ungrazed areas, communities with *P*. *cancellata* (CFSs) had a significantly 67% lower species richness than communities without this plant (WFSs). The decreases in the reciprocal of the Gini-Simpson index in CFSs was not significant and had a value of 38%.

**Figure 2:**
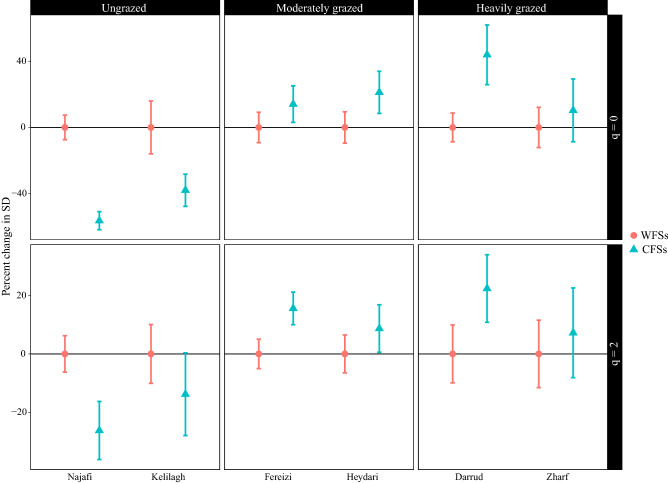
The coverage-based comparison of Hill phylogenetic diversity of the plant communities containing (CFSs) and without (WFSs) *Phlomis cancellata* in different grazing conditions. The phylogenetic richness (q = 0) and phylogenetic diversity of dominant species (q = 2) were reported in this study. PD = phylogenetic diversity. The results are reported as the percentage change in PD of CFSs comparing to that of WFSs.

The phylogenetic diversity results showed that, in the ungrazed sites, CFSs had a 56% (the Najafi) and 38% (the Kelilagh) lower phylogenetic richness (q = 0) significantly compared to WFSs (Fig. 2). Considering the phylogenetic diversity of the dominant species (i.e., q = 2), CFSs had lower phylogenetic diversity than WFSs, with values of 26% (the Najafi) and 14% (the Kelilagh). The decrease in the phylogenetic diversity of dominant plants was significant in the Najafi and nonsignificant in the Kelilagh. Thus, on average, CFSs had a significantly 47% lower phylogenetic richness than WFSs in ungrazed sites. The decrease in the phylogenetic diversity of dominant species in CFSs had an average value of 20%.

In the moderately grazed sites, CFSs had an 11% higher species richness in the Fereizi and 10% higher in the Heydari than WFSs. This increase in the species richness was not significant in these sites (Fig. 1). On average, the species richness of CFSs in moderately grazed sites had a non-significantly 11% higher species richness than WFSs. Considering the reciprocal of the Gini-Simpson index, CFSs of the Fereizi and Heydari had higher diversity than WFSs with a value of 23% and 45%, respectively. This finding was nonsignificant in the Fereizi but significant in the Heydari (Fig. 1). The average increase in the reciprocal of the Gini-Simpson index in CFSs was 34% compared to WFSs in the moderately grazed sites.

In the moderately grazed areas, CFSs had higher phylogenetic richness than WFSs, with a value of 14% in the Fereizi and 21% in the Heydari. This phylogenetic richness increase was significant in neither of the sites (Fig. 2). Thus, on average CFSs had 18% higher phylogenetic richness than WFSs in the moderately grazed sites. Considering the phylogenetic diversity at the level of dominant species, the increase in diversity of CFSs for the Fereizi and Heydari was 16% and 9%, respectively. This finding was significant in the Fereizi and not significant in the Heydari (Fig. 2). Therefore, the average phylogenetic diversity of dominant species in CFSs was 12% higher compared to WFSs in moderately grazed areas.

In the heavily grazed sites, in the Darrud, CFSs had 37% higher species richness and 8% higher the reciprocal of the Gini-Simpson index than WFSs. These increases were not significant (Fig. 1). In the Zharf, species richness (8%) and the reciprocal of the Gini-Simpson index (17%) were higher in CFSs than WFSs. In this site, the difference between CFSs and WFSs was not significant (Fig. 1). On average, CFSs had non-significantly 23% higher species richness than WFSs in the heavily grazed sites. For the reciprocal of the Gini-Simpson index, the difference between CFSs and WFSs was not significant and CFSs on average had 11% higher diversity.

In the heavily grazed sites, CFSs had 44% (the Darrud) and 10% (the Zharf) higher phylogenetic richness (q = 0) than WFSs. The increase in phylogenetic richness was significant in the Darrud but not significant in the Zharf (Fig. 2). Considering the phylogenetic diversity at the level of dominant species (q = 2), CFSs had higher diversity than WFSs. This difference was significant in the Darrud and not significant in the Zharf with 22% and 7% values, respectively. On average, CFSs had 27% higher phylogenetic richness than WFSs in the heavily grazed sites. The increase in the phylogenetic diversity of dominant species in CFSs had an average value of 15%.

**Figure 3:**
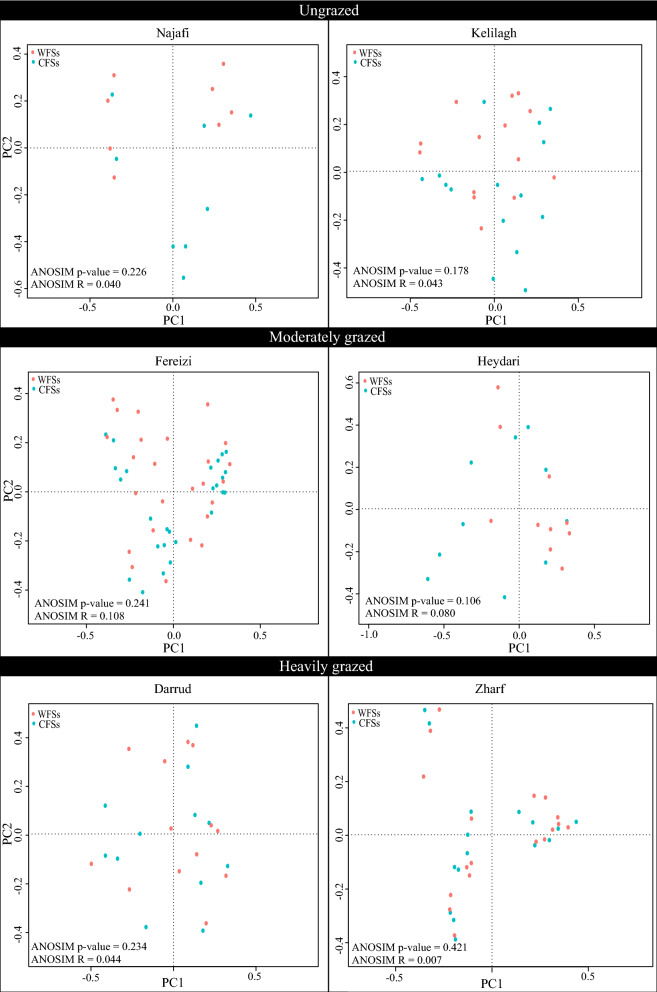
Transformation-based principal component analysis and analysis of similarity (ANOSIM) results showing the species composition differences in plant communities containing (CFSs) and without (WFSs) *Phlomis cancellata* in six sites.

No significant difference was detected among the species compositions of CFSs and WFSs in the six sites. Figure 3 presents the tb-PCA and ANOSIM results for each of the study areas.

### *Euphorbia boissieriana* and *E. microsciadia*

**Figure 4:**
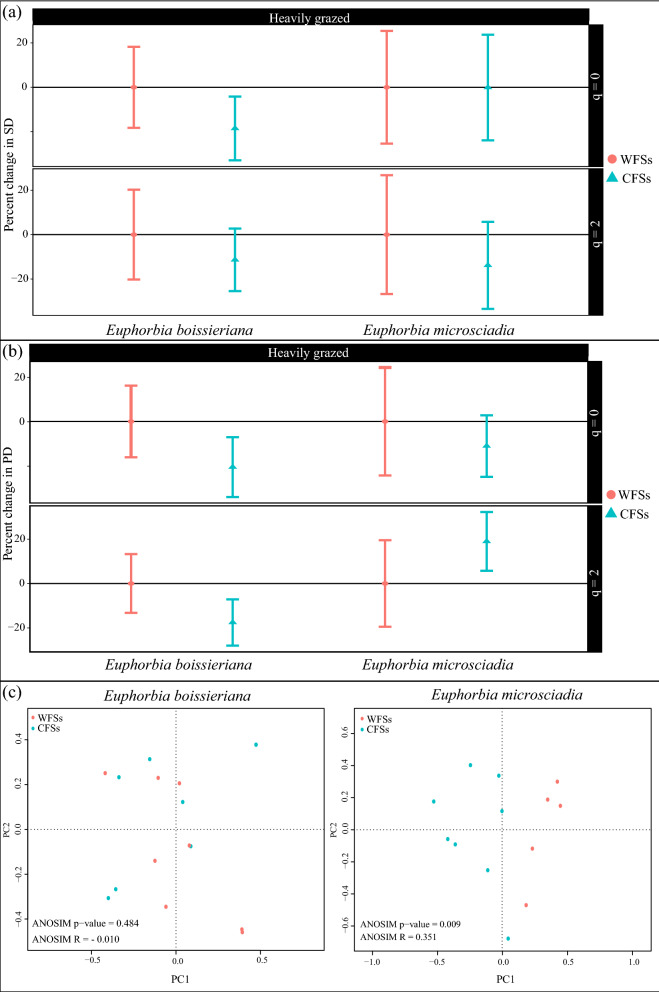
The coverage-based comparison of (**a**) species and (**b**) phylogenetic diversity of communities containing (CFSs) and without (WFSs) *Euphorbia boissieriana* and *Euphorbia microsciadia*. (**c**) Transformation-based principal component analysis and analysis of similarity (ANOSIM) results showing the species composition differences in CFSs and WFSs.

For *E. boissieriana*, in the Darrud—a heavily grazed site, CFSs had 19% lower species richness and 11% lower the reciprocal of the Gini-Simpson index than WFSs. The difference in species diversity was significant at neither of the levels (Fig. 3). Considering the phylogenetic diversity indices, the phylogenetic diversity of CFSs was 20% and 18% lower than WFSs for q = 0 and 2, respectively. This result was not significant at any of the q levels (Fig. 4).

For *E. microsciadia*, in the Boghmech (a heavily grazed site), CFSs had 0.1% (species richness) and 13% (the reciprocal of the Gini-Simpson index) lower diversity compared to WFSs. The difference in species diversity was not significant at the both levels (Fig. 4). Considering the phylogenetic diversity, CFSs had lower phylogenetic richness than WFSs. This difference was not significant and had a value of 11%. On the contrary, a 19% non-significantly increase in phylogenetic diversity of dominant species was observed in CFSs compared to WFSs.

No significant difference was detected between the species compositions of CFSs and WFSs for *E. boissieriana*. However, there was a significant difference between the species composition of the two communities for *E. microsciadia*. Figure 4 shows the species and phylogenetic diversity results along with tb-PCA results for CFSs and WFSs.

### *Seseli transcaucasicum*

**Figure 5:**
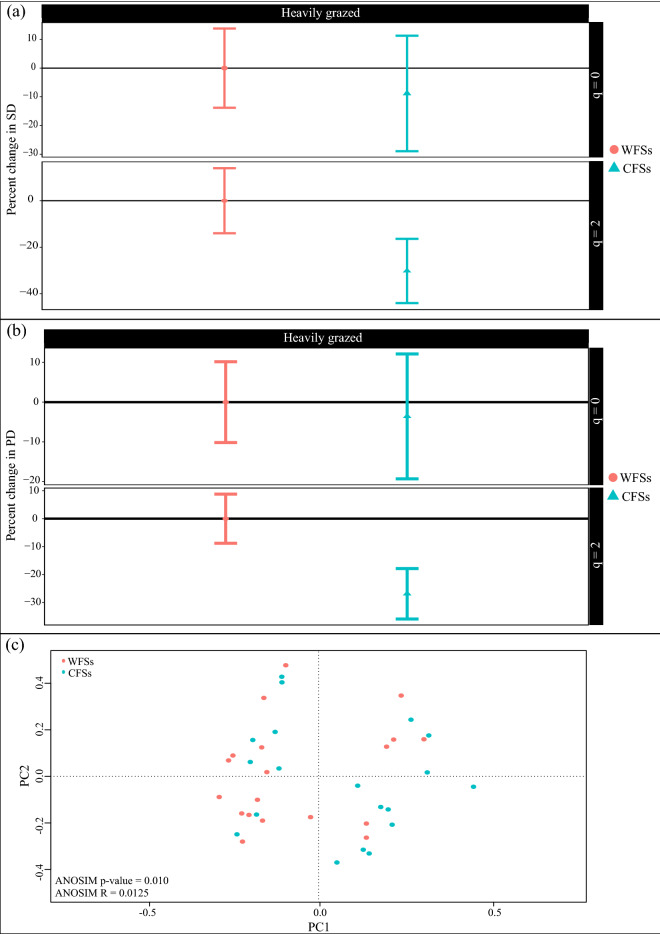
The coverage-based comparison of (**a**) species and (**b**) phylogenetic diversity of communities containing (CFSs) and without (WFSs) *Seseli transcaucasicum*. (**c**) Transformation-based principal component analysis and analysis of similarity (ANOSIM) results showing the species composition differences in CFSs and WFSs.

In the Arabchah—a heavily grazed site, CFSs had 9% lower species richness and 30% lower the reciprocal of the Gini-Simpson index than WFSs. This difference in species diversity was only significant at the level of dominant species (q = 2) (Fig. 5a). The phylogenetic richness (q = 0) of CFSs was non-significantly 3% lower than WFSs. Phylogenetic diversity of dominant species in CFSs was significantly 27% lower than WFSs (Fig. 5b). There was a significant difference between the species compositions of CFSs and WFSs (Fig. 5c). Table 2 summarizes our results.

**Table 2:** Summary of the effects of four unpalatable species on neighboring communities.

Species name	Site	Grazing condition	SD		PD		SC
			q = 0	q = 2	q = 0	q = 2	
*Phlomis cancellata*	Keleilagh	Ungrazed	WFSs*	WFSs^ns^	WFSs*	WFSs^ns^	ns
	Najafi	Ungrazed	WFSs*	WFSs*	WFSs*	WFSs*	ns
	Fereizi	Mod. grazing	CFSs^ns^	CFSs*	CFSs^ns^	CFSs*	ns
	Heydari	Mod. grazing	CFSs^ns^	CFSs*	CFSs^ns^	CFSs^ns^	ns
	Zharf	Hea. grazing	CFSs^ns^	CFSs^ns^	CFSs^ns^	CFSs^ns^	ns
	Darrud	Hea. grazing	CFSs^ns^	CFSs^ns^	CFSs*	CFSs*	ns
*Euphorbia microsciadia*	Boghmech	Hea. grazing	WFSs^ns^	WFSs^ns^	WFSs^ns^	WFSs^ns^	*
*Euphorbia boissieriana*	Darrud	Hea. grazing	WFSs^ns^	WFSs^ns^	WFSs^ns^	WFSs^ns^	ns
*Seseli transcaucasicum*	Arabchah	Hea. grazing	WFSs^ns^	WFSs*	WFSs^ns^	WFSs*	*

For SD and PD, a community with a higher value is reported in the table.

Abbreviations: *SD* species diversity; *PD* phylogenetic diversity; *SC* species composition; *CFSs* contain focal species; *WFSs* without focal species;

*ns* no significant difference. *Indicated a significant difference at a 5% significance level.

## Discussion

It has previously been reported that unpalatable species protect their neighbors from grazing^4^, our results indicated the type of association effect depends on the unpalatable species.

### *Phlomis cancellata*: associational defense

Our results revealed that *P*. *cancellata* had the same effect on neighboring plants in areas with the same grazing condition. Our results indicated *P. cancellata* helped neighbors to compensate for grazing. As a result, CFSs had higher biodiversity than WFSs. However, neighboring with this plant had negative impacts in the ungrazed areas. The canopy of *P*. *cancellata* creates a shady condition that might be unfavorable in ungrazed conditions. Similar results were reported for *Urtica thunbergiana*. This species was a facilitator in the grazing conditions but a competitor in ungrazed areas^2^. It was also reported that *Juncus effusus* increased the diversity of neighboring communities in a grazed area and competed with neighbors in an ungrazed site. However, *J. effusus* affected the species composition of neighboring communities in the grazed site^37^. The relation of *P. cancellata* with neighbors in ungrazed conditions might be a competitive interaction. In the presence of large herbivores, species benefit from associating with this plant. This finding suggested that biotic stresses affect the plant–plant interactions. A similar condition was also reported for *Filipendula ulmaria*^38^.

### *Euphorbia boissieriana* and *E. microsciadia*: neighbor contrast susceptibility

Our results revealed that for both studied *Euphorbia* species, WFSs showed non-significantly higher diversity than CFSs. These results suggested a case of neighbor contrast susceptibility. The phylogenetic diversity results implied that not every species were able to benefit from neighboring *E*. *boissieriana* or *E*. *microsciadia*, except for those species from relative taxa showing a similar evolutionary history. These two species, through biotic interaction, do not allow every plant to grow nearby. Allelopathy, which is common among *Euphorbia* species^39–42^, might be that biotic interaction. The allelopathic effect of *E*. *microsciadia* was possibly stronger than that of *E*. *boissieriana*. The species composition of communities with *E*. *microsciadia* was significantly different from communities without this plant. A study on *E. schickendatzii* reported that species diversity was higher in the plant communities containing *E. schickendatzii* when compared with communities without this plant. Also, the species composition of the two communities was significantly different^12^.

### *Seseli transcaucasicum*: neighbor contrast susceptibility

The species composition of CFSs was different from that of WFSs. Furthermore, communities with *S*. *transcaucasicum* showed a lower species diversity and phylogenetic diversity than communities without this plant. Similar to the studied *Euphorbia* species, *S*. *transcaucasicum* induced neighbor contrast susceptibility in neighboring communities. Similar to *E*. *boissieriana* and *E*. *microsciadia* but with a higher magnitude, few species from relative lineages could grow near *S*. *transcaucasicum*. Gao et al.^11^ suggested that range expansion of poisonous species can result in an altered community structure.

### Comparing the four species: herbivore grazing hierarchy

Herbivores make foraging decisions at different spatial scales simultaneously^43,44^. The variation in associational effects among the studied species was due to herbivore different foraging decisions at different spatial scales (within- or between-patches). Our finding suggested that grazers selectively choose another patch when facing *P*. *cancellata*. On the other hand, they choose from plant individuals when there was a poisonous plant (i.e., *E. boissieriana*, *E. microsciadia*, and *S. transcaucasicum*) in a selected patch. Furthermore, comparing different grazing levels, it can be declared that grazing intensity may not have a strong effect on the foraging decision of sheep and goats in response to unpalatable species.

### Limitations

This study is an observational and not experimental research. Considering this limitation is important when extending our results to the other areas. Regarding our data, the Fereizi plots were 3 m × 3 m quadrats. We did not compare the results among the sites to avoid any biases that come from this limitation. Also, we used another data from a moderately grazed site to strengthen our inferences. For *S*. *transcaucasicum*, data from one site was available. As a result, we could not account for the variation among the sites. All study areas have similar climatic conditions (i.e., semi-arid climate). Therefore, we could not test whether different climatic conditions affect the impacts of unpalatable species on neighboring communities.

## Conclusions

We have observed that the unfavorable microhabitat of *P*. *cancellata* in ungrazed areas could become a possible shelter in grazing conditions. However, neighboring with a poisonous species could increase the herbivore damage on a plant community. We encourage to perform an experimental study on the effects of these studied species on neighboring communities. We have used a coverage-based approach for comparing biodiversity between CFSs and WFSs. We suggest using this method for future studies dealing with plant community comparisons.

## Supplementary Information

Supplementary Information.

## Data Availability

The data regarding this study is presented in Supplementary Information.
