# Supplementary material for: Unpalatable plants induce a species-specific associational effect on neighboring communities
Source: Sci Rep. 2021 Jul 13;11:14380. doi: 10.1038/s41598-021-93698-4 (PMC8277879; doi:10.1038/s41598-021-93698-4)
Supplement: Supplementary file 1 — Supplementary Information. [file 41598_2021_93698_MOESM1_ESM.pdf]

Euphorbia boissieriana
Darrud

CFS

Acantholimon_erinaceum	1	0	0	0	0	0
Acroptilon_repens	0	0	0	0	0	0
Alhagi_maurorum	0	0	1	0	0	1
Allium_tenuicaule	0	0	0	0	0	1
Alyssum_desertorum	0	0	0	0	0	0
Asperula_glomerata	0	0	0	0	0	0
Astragalus_callainus	2	0	1	0	0	1
Astragalus_campylotrichus	0	2	0	0	2	0
Astragalus_catacamptus	0	0	0	0	0	0
Astragalus_esferayenicus	0	0	0	1	0	0
Astragalus_lycioides	0	0	0	0	0	0
Astragalus_verus	0	1	0	2	1	0
Barbarea_plantaginea	0	2	0	0	0	0
Boissiera_squarrosa	0	0	0	0	0	0
Bromus_danthoniae	0	0	4	9	0	4
Bromus_tectorum	6	0	0	18	0	0
Bupleurum_falcatum	0	1	0	0	1	0
Calamagrostis_pseudophragmites	0	0	0	0	0	0
Capparis_spinosa	0	0	0	0	0	0
Centaurea_virgata	0	0	1	0	0	1
Cerastium_inflatum	0	0	0	0	0	0
Ceratocephala_falcata	2	0	0	0	0	0
Ceratocephala_testiculata	5	0	0	0	0	0
Chaerophyllum_khorossanicum	0	0	0	0	0	0
Cichorium_intybus	0	2	0	0	2	0
Cirsium_congestum	0	0	1	0	0	1
Cousinia_concolor	0	1	1	0	1	1
Crambe_kotschyana	0	0	0	0	0	0
Cymbolaena_griffithii	0	0	0	0	0	0
Dactylis_glomerata	0	0	0	2	0	0
Dianthus_polylepis	0	0	0	0	0	0
Echinophora_platyloba	0	2	0	0	2	1
Echinops_orientalis	0	0	0	0	1	0
Elwendia_afghanica	5	0	3	0	0	3
Elwendia_chaerophylloides	0	0	0	0	0	0
Eryngium_billardierei	0	0	0	0	0	0
Eryngium_bungei	0	0	0	0	0	0
Euphorbia_bungei	0	1	0	0	1	0
Euphorbia_szovitsii	0	0	0	2	0	0
Gagea_chomutowae	0	0	0	0	0	0
Galium_aparine	0	0	1	0	0	1
Galium_spurium	0	0	0	0	0	0
Galium_tricornutum	6	0	0	0	0	0
Geranium_rotundifolium	0	0	0	0	0	0
Gundelia_tournefortii	1	1	0	0	1	0
Hymenocrater_platystegius	0	0	0	0	0	0
Hypecoum_pendulum	0	0	0	0	0	0
Hypericum_perforatum	0	0	0	0	0	0
Iris_kopetdagensis	0	0	0	0	0	0
Ixiolirion_tataricum	0	0	0	0	0	0

Lactuca_orientalis	0	0	0	0	0	0
Lactuca_serriola	0	0	0	2	0	0
Lamium_amplexicaule	0	0	0	0	0	0
Melica_persica	0	0	0	0	0	0
Melilotus_officinalis	0	0	0	0	0	0
Mentha_longifolia	0	0	0	0	0	0
Muscari_neglectum	0	0	1	0	0	1
Neotorularia_aculeolata	0	0	0	0	0	0
Nepeta_bracteata	0	0	0	0	0	0
Noaea_mucronata	0	1	0	2	1	0
Onosma_longiloba	0	0	0	0	0	0
Oxytropis_kuchanensis	0	0	0	0	0	0
Pachypterygium_brevipes	0	0	0	0	0	0
Parietaria_judaica	0	0	0	0	0	0
Perovskia_abrotanoides	0	0	0	0	0	0
Phlomis_cancellata	0	0	0	0	0	0
Pimpinella_affinis	0	0	0	0	0	0
Pimpinella_khayyamii	0	3	0	0	3	0
Poa_bulbosa	0	0	3	0	0	3
Poa_pratensis	4	3	0	0	3	0
Polygonum_afghanicum	0	0	0	0	0	0
Polygonum_arenastrum	3	0	0	0	0	0
Polygonum_aviculare	0	0	0	7	0	0
Polygonum_thymifolium	0	0	0	0	0	0
Roemeria_hybrida	0	0	0	0	0	0
Rosa_persica	0	0	0	0	0	0
Scandix_stellata	0	0	7	0	0	4
Scorzonera_raddeana	0	0	0	0	0	0
Stellaria_media	0	0	0	0	0	0
Tanacetum_parthenium	0	0	0	0	0	0
Taraxacum_officinale	0	0	0	0	1	0
Thalictrum_isopyroides	0	0	0	0	0	0
Trichophorum_pumilum	0	0	0	0	0	0
Tulipa_micheliana	0	0	0	0	0	0
Verbascum_songaricum	1	0	0	0	0	0
Veronica_anagallisaquatica	0	0	0	0	0	0
Vicia_subvillosa	0	0	0	0	0	0
Vincetoxicum_pumilum	0	0	1	0	0	2
Vulpia_persica	0	0	0	0	0	0
Ziziphora_tenuior	0	0	0	3	0	0

0	0
0	0
0	0
0	5
0	0
0	0
0	0
0	0
0	0
0	0
0	0
0	0
0	0
0	0
0	0
0	0
0	0
0	0
0	0
0	0
0	0
0	0
0	0
0	0
0	0
0	0
6	0
0	1
0	0
0	0
0	0
0	4
0	0
0	0
0	0
0	1
0	0
0	0
0	0
0	0
5	0
0	0
0	0
0	0
0	0
0	4
0	8
0	0
0	0
0	0

0	0
0	0
0	0
1	0
0	0
0	0
0	0
0	3
0	0
2	0
2	0
3	0
0	0
0	0
0	0
1	0
1	0
0	0
0	0
0	0
0	3
0	0
0	0
0	0
0	0
1	0
0	0
0	2
0	0
0	3
0	0
0	0
0	0
1	0
0	0
0	0
0	2
0	2
0	0
0	0

WFS

Acantholimon_erinaceum	0	0	0	0	0
Acroptilon_repens	0	0	0	0	0
Alhagi_maurorum	0	2	0	0	2
Allium_tenuicaule	0	0	0	0	0
Alyssum_desertorum	0	0	0	2	0
Asperula_glomerata	0	4	3	0	4
Astragalus_callainus	0	0	0	1	0
Astragalus_campylotrichus	0	0	0	0	0
Astragalus_catacamptus	0	0	0	0	0
Astragalus_esferayenicus	0	0	0	0	0
Astragalus_lycioides	0	0	0	1	0
Astragalus_verus	1	0	1	1	0
Barbarea_plantaginea	0	0	0	0	0
Boissiera_squarrosa	0	0	0	13	0
Bromus_danthoniae	0	0	0	12	0
Bromus_tectorum	0	0	0	20	0
Bupleurum_falcatum	0	0	0	0	0
Calamagrostis_pseudophragmites	0	0	0	0	0
Capparis_spinosa	0	0	0	0	0
Centaurea_virgata	1	1	1	3	1
Cerastium_inflatum	0	0	0	0	0
Ceratocephala_falcata	0	0	0	0	0
Ceratocephala_testiculata	0	0	0	0	0
Chaerophyllum_khorossanicum	0	0	0	0	0
Cichorium_intybus	0	2	0	0	2
Cirsium_congestum	0	0	0	0	0
Cousinia_concolor	2	4	0	0	4
Crambe_kotschyana	0	0	0	0	0
Cymbolaena_griffithii	0	0	0	0	1
Dactylis_glomerata	0	0	0	0	0
Dianthus_polylepis	0	0	2	0	0
Echinophora_platyloba	0	0	0	0	0
Echinops_orientalis	0	0	0	0	0
Elwendia_afghanica	0	0	0	0	0
Elwendia_chaerophylloides	0	3	0	0	3
Eryngium_billardierei	0	0	0	0	0
Eryngium_bungei	0	0	1	0	0
Euphorbia_bungei	0	0	0	0	0
Euphorbia_szovitsii	3	4	2	0	4
Gagea_chomutowae	0	3	0	0	3
Galium_aparine	0	0	0	0	0
Galium_spurium	0	2	5	0	2
Galium_tricornutum	10	0	0	0	0
Geranium_rotundifolium	0	0	0	0	0
Gundelia_tournefortii	0	2	0	0	2
Hymenocrater_platystegius	0	0	0	0	0
Hypecoum_pendulum	0	0	0	0	0

Hypericum_perforatum	0	0	0	0	0
Iris_kopetdagensis	0	0	0	0	0
Ixiolirion_tataricum	0	1	0	0	1
Lactuca_orientalis	3	0	4	1	0
Lactuca_serriola	0	0	0	0	0
Lamium_amplexicaule	0	0	0	0	0
Melica_persica	0	0	3	0	0
Melilotus_officinalis	0	0	0	0	0
Mentha_longifolia	0	0	0	0	0
Muscari_neglectum	0	0	0	0	0
Neotorularia_aculeolata	0	0	0	0	0
Nepeta_bracteata	0	0	0	0	0
Noaea_mucronata	0	0	0	0	0
Onosma_longiloba	0	0	0	0	0
Oxytropis_kuchanensis	0	0	0	0	0
Pachypterygium_brevipes	0	0	0	0	0
Parietaria_judaica	0	0	2	0	0
Perovskia_abrotanoides	0	0	0	0	0
Phlomis_cancellata	0	0	0	3	0
Pimpinella_affinis	0	0	0	0	0
Pimpinella_khayyamii	0	0	0	0	0
Poa_bulbosa	0	0	4	0	0
Poa_pratensis	0	0	0	0	0
Polygonum_afghanicum	0	0	0	0	0
Polygonum_arenastrum	0	0	0	3	0
Polygonum_aviculare	0	0	5	0	0
Polygonum_thymifolium	0	0	0	0	0
Roemeria_hybrida	0	0	0	0	0
Rosa_persica	1	0	0	0	0
Scandix_stellata	0	0	2	0	0
Scorzonera_raddeana	0	0	0	0	0
Stellaria_media	0	0	0	0	0
Tanacetum_parthenium	0	0	0	0	1
Taraxacum_officinale	0	0	0	0	0
Thalictrum_isopyroides	0	0	0	0	0
Trichophorum_pumilum	0	0	0	0	0
Tulipa_micheliana	0	0	0	1	0
Verbascum_songaricum	0	0	0	0	0
Veronica_anagallisaquatica	8	0	0	0	0
Vicia_subvillosa	0	0	0	0	0
Vincetoxicum_pumilum	0	1	0	0	1
Vulpia_persica	0	0	0	0	0
Ziziphora_tenuior	0	0	0	3	0

0	0	0
0	2	0
0	0	1
0	0	0
0	0	0
0	0	0
0	0	3
0	0	4
0	0	1
0	0	0
0	0	0
2	1	0
0	0	0
0	0	0
0	2	0
0	0	0
0	1	0
0	1	2
0	0	1
0	0	0
3	0	0
0	0	0
0	0	0
0	0	2
0	0	0
1	0	0
2	0	0
0	0	0
0	0	0
0	0	0
0	0	0
0	0	0
0	0	0
0	0	0
0	0	0
0	0	0
2	0	0
0	0	0
0	0	0
0	0	0
0	0	0
0	0	0
0	0	0
0	0	6
0	0	0
0	0	0
0	0	0

0	6	0
0	0	2
0	0	0
0	0	0
1	0	0
0	1	0
0	0	0
0	1	1
0	2	0
0	0	0
0	0	0
0	1	0
3	0	0
0	0	0
0	0	0
2	0	0
0	0	0
1	0	0
1	0	0
0	0	0
0	0	0
7	0	0
0	0	0
0	0	0
0	0	0
0	0	0
0	4	0
0	0	4
0	0	0
2	0	0
0	0	0
0	0	3
0	3	0
0	0	0
2	0	0
0	1	0
0	0	0
0	0	0
0	0	0
0	0	0
0	0	0
0	0	7
0	0	0

Euphorbia microsciadia Boghmech

CFS

Acanthophyllum_glandulosum	0	0	0	90	0	0	0	0	0	15
Allium_monophyllum	0	0	0	0	0	0	0	0	0	0
Alyssum_desertorum	5	1	25	0	0	0	0	0	0	0
Alyssum_sp_	0	0	0	0	0	0	5	0	0	0
Astragalus_verus	0	0	0	0	0	0	0	0	20	0
Astragalus_sp_	0	0	0	0	0	0	0	0	5	0
Bromus_tectorum	0	0	6	0	0	0	0	0	0	0
Calamagrostis_pseudophragmites	0	0	60	0	0	0	0	0	0	0
Carex_minor	0	0	0	0	0	0	0	0	15	0
Ceratocephala_testiculata	0	0	0	0	3	0	3	0	5	0
Cirsium_sp_	0	15	35	0	0	0	15	20	35	0
Convolvulus_arvensis	0	0	3	0	0	0	0	0	0	0
Convolvulus_lineatus	0	0	0	0	0	0	0	0	0	4
Cousinia_microcarpa	0	0	0	0	0	0	10	0	35	0
Cousinia_multiloba	0	0	0	0	0	10	30	0	45	0
Eryngium_bungei	0	0	25	0	0	0	0	0	0	0
Euphorbia_bungei	0	0	0	0	0	0	0	5	15	0
Iris_sp_	0	0	0	0	0	0	0	0	0	0
Lactuca_sp_	0	0	25	0	0	0	0	0	0	0
Lotus_corniculatus	0	0	15	0	0	0	0	0	0	0
Mentha_longifolia	0	0	10	0	0	0	0	0	0	0
Onobrychis_cornuta	0	0	0	0	50	30	160	30	95	5
Poa_bulbosa	20	0	20	0	0	6	6	10	42	0
Polygonum_afghanicum	0	0	0	0	10	10	30	0	0	0
Polygonum_arenastrum	0	0	0	0	0	0	0	0	30	0
Polygonum_aviculare	0	0	15	0	0	0	0	0	0	0
Prunus_spinosissima	0	50	50	0	0	0	0	0	0	0
Rosa_beggeriana	0	0	60	0	0	0	0	0	0	0
Rosa_canina	0	0	90	0	0	0	0	0	0	0
Rumex_sp_	0	0	25	0	0	0	0	0	0	0
Scorzonera_sp_	0	0	0	0	0	0	0	10	10	0
Stachys_sp_	0	0	0	0	0	0	0	0	10	0
Tanacetum_turcomanicum	0	0	0	0	0	10	10	0	0	0
Taraxacum_brevirostre	0	0	0	0	16	0	16	0	8	0
Taraxacum_iranicum	0	0	10	0	0	0	0	0	0	0
Thymus_sp_	0	0	0	0	0	0	50	0	0	0
Valeriana_sisymbriifolia	0	0	0	0	0	0	0	0	0	0
Verbascum_sp_	60	30	90	0	0	0	0	0	0	0
Veronica_biloba	6	0	13	0	4	0	4	0	5	0

WFS

Acanthophyllum_glandulosum	0	0	0	0	0	0	0	0
Allium_monophyllum	0	0	0	0	0	0	0	2
Alyssum_desertorum	9	0	0	0	0	0	0	0
Alyssum_sp_	0	0	5	0	0	0	0	0
Astragalus_verus	0	0	0	0	0	20	0	0
Astragalus_sp_	0	0	0	0	0	5	0	0
Bromus_tectorum	0	6	0	0	0	0	0	0
Calamagrostis_pseudophragmites	20	0	0	0	0	0	0	0
Carex_minor	0	0	0	0	0	0	0	15
Ceratocephala_testiculata	0	0	0	0	5	0	0	0
Cirsium_sp_	0	0	0	15	5	0	0	0
Convolvulus_arvensis	0	3	0	0	0	0	0	0
Convolvulus_lineatus	0	0	0	0	0	0	6	31
Cousinia_microcarpa	0	0	0	10	35	0	0	0
Cousinia_multiloba	0	0	20	0	0	15	20	30
Eryngium_bungei	5	0	0	0	0	0	0	0
Euphorbia_bungei	0	0	0	0	10	0	0	0
Iris_sp_	0	0	0	0	0	0	75	75
Lactuca_sp_	5	20	0	0	0	0	0	0
Lotus_corniculatus	0	15	0	0	0	0	0	0
Mentha_longifolia	0	10	0	0	0	0	0	0
Onobrychis_cornuta	0	0	40	40	5	0	25	45
Poa_bulbosa	0	0	0	0	6	16	0	0
Polygonum_afghanicum	0	0	0	10	0	0	0	0
Polygonum_arenastrum	0	0	0	0	0	25	0	0
Polygonum_aviculare	0	15	0	0	0	0	0	0
Prunus_spinosissima	0	0	0	0	0	0	0	0
Rosa_beggeriana	0	0	0	0	0	0	0	0
Rosa_canina	90	0	0	0	0	0	0	0
Rumex_sp_	0	25	0	0	0	0	0	0
Scorzonera_sp_	0	0	0	0	0	0	0	0
Stachys_sp_	0	0	0	0	10	0	0	0
Tanacetum_turcomanicum	0	0	0	0	0	0	0	0
Taraxacum_brevirostre	0	0	0	0	2	6	0	0
Taraxacum_iranicum	0	10	0	0	0	0	0	0
Thymus_sp_	0	0	30	20	0	0	0	0
Valeriana_sisymbriifolia	0	0	0	0	0	0	0	20
Verbascum_sp_	0	0	0	0	0	0	0	0
Veronica_biloba	4	0	0	0	0	5	0	0

Phlomis cancellata
Darrud

CFS

Acantholimon_erinaceum	1	0	1	0	0	1	0.5	0	2	5	1.75	1
Acanthophyllum_crassinodum	0	0	0	0	0	0	0	0	0	0	0	0
Acanthophyllum_glandulosum	0	1	0	0	0	0	0.16	0.25	0	0	0	0
Allium_kuhsorkhense	0	1	0	0	0	0	2	0	0	0	0	0
Allium_tenuicaule	0	0	0	0	3	0	0	0	0	0	0	0
Alyssum_desertorum	0	0	0	0	0	0	0	0	0	0	1	0
Alyssum_stapfii	0	0	0	0	0	0	0	0	0	0	0	0
Arrhenatherum_kotschyi	0	0	0	0	0	0	0.5	0	0	0	0	0
Asperula_glomerata	1	0	0	0	0	1	0	0	0	0	0	0
Astragalus_citrinus	0	0	0	0	0	0	0	0.25	0	0	0	0
Astragalus_masanderanus	0	0	1	0	0	1	0	0	0	0	0	0
Astragalus_verus	0	0	0	0	1	0	0.08	0	0	0	1.5	1
Biebersteinia_multifida	0	0	0	0	0	0	0	0	0	0	0	1
Boissiera_squarrosa	0	0	0	0	0	0	0	0	4	0	0	0
Bromus_danthoniae	0	3	0	0	6	5	10	0	0	0	0	0
Bromus_oxyodon	0	0	0	0	0	0	0	0	0	0	0	0
Bromus_tectorum	0	0	0	6	0	0	4.25	0.5	0	0	0	0.5
Callipeltis_cucullaria	0	0	0	0	0	0	0	0	0	0	0	0
Centaurea_virgata	0	0	0	0	0	0	0	0	0	0	0	0
Cerastium_inflatum	0	0	0	0	0	0	0	0	0	0.75	0.75	0
Ceratocarpus_arenarius	0	0	0	0	0	0	0	0	0	0	0	1
Ceratocephala_falcata	0	0	0	0	0	0	0	0	30	2	0	1
Ceratocephala_testiculata	0	0	0	0	0	0	0	0	0	0	0	0
Cirsium_congestum	0	0	0	0	3	0	0	1	0	0	0	0
Cirsium_turkestanicum	0	0	0	0	0	0	0	0	0	2	0	0
Colchicum_robustum	0	0	0	0	0	0	0.04	0	0	0	0	0
Cousinia_concolor	2	0	0	0	3	0	1	0	0	1	0	0
Cousinia_multiloba	0	0	2	0	0	0	0	0	0	0	3	0
Cymbolaena_griffithii	0	0	0	0	0	0	0	2	0	0	0	0
Drabopsis_verna	0	0	0	0	0	0	0	0	1	0	0	0
Elwendia_afghanica	4	4	0	0	0	0	0	0	0	0	0	0
Elymus_hispidus	0	0	0	0	0	0	0	0	0	0	0	0
Eranthis_longistipitata	0	0	2	0	0	0	0	0	0	0	0	0
Eremurus_spectabilis	3	1	0	0	0	0	0.5	1.25	0	1.5	0	0.25
Eremurus_stenophyllus	0	0	0	0	0	0	0	0	0	0	1	0
Euphorbia_microsciadia	0	0	0	0	0	0	0	0	0	0	0	0.5
Euphorbia_szovitsii	0	0	0	0	0	0	2	0	0	0	0	0.5
Fallopia_convolvulus	0	0	0	0	0	0	0	1.75	0	0	0	0
Gagea_chomutowae	0	0	0	0	0	0	0	0	0	0	0	0
Gagea_gageoides	0	0	0	0	0	0	0	0	0	0	0	3
Gagea_kunawurensis	0	0	6	0	0	0	0	0	0	1.25	0	0
Gagea_reticulata	0	0	0	0	0	0	3	0	0	0	0	0
Gagea_setifolia	0	0	0	6	0	0	4	0	8	0	0	0
Galium_aparine	0	0	0	0	4	0	0	0	0	0	0	0
Galium_humifusum	0	0	0	0	0	0	0	0	0	0	0	1.25
Galium_tricornutum	0	0	0	0	0	0	3	0.75	0	5	0	0
Holosteum_glutinosum	0	0	0	0	0	0	0	0	0	0	0	0
Hypericum_scabrum	0	0	0	0	0	0	0.06	0	0	0	0	0
Ixiolirion_tataricum	0	2	0	0	0	0	0	0	0	0	0	0.5
Lactuca_orientalis	0	0	0	0	0	0	0	0	0	1	0	0
Lactuca_serriola	0	0	0	0	0	0	0	0	0	0	0	0.5
Lappula_sinaica	0	0	3	0	0	0	0	0.25	0	0	0	0
Lepyrodiclis_stellarioides	0	0	0	0	0	0	0	0	0	0	3.5	0
Melica_persica	0	0	0	0	0	0	0.5	0	0	0	0	0
Nepeta_bracteata	0	0	0	0	0	0	0	0	0	1	0	0
Noaea_mucronata	0	0	0	0	0	0	0	0	0	0	0.5	0
Onosma_dichroantha	0	0	0	0	0	0	0	0	0	0	0	0
Onosma_longiloba	0	0	0	0	0	0	0	0	0	0	0	0
Papaver_dubium	0	0	0	0	0	0	0	0	0	0	0.75	0
Perovskia_abrotanoides	0	0	0	0	0	0	0	0	0	0	0	0
Pimpinella_affinis	0	0	2	0	0	0	0	0	0	1	1	0
Poa_bulbosa	0	0	0	0	0	0	0	0	0	0	0	0
Poa_pratensis	0	0	0	4	0	3	0.5	0.5	0	0	0	1
Polygonum_aviculare	0	4	0	0	0	0	0	0	0	0	0	0
Polygonum_polycnemoides	0	0	0	2	0	0	0	0	0	0	0	0
Polygonum_thymifolium	0	0	0	0	0	0	0	0	2	0	0	0
Rheum_khorasanicum	0	0	0	0	0	0	0	0.75	9	0	0	0
Rumex_tianschanicus	3	0	0	0	0	0	0.24	0	0	0	0	0
Salvia_chloroleuca	0	0	0	0	0	0	0	0	0	1	0	0
Scandix_stellata	0	0	1	0	0	0	0.5	0	1	0	0	0
Scrophularia_crassipedunculata	0	0	0	0	0	0	0	0	0	0	4	0
Scrophularia_variegata	0	0	0	0	0	0	0	0	0	0	0	0
Silene_latifolia	0	0	0	0	0	0	0.5	0	0	0	0	0
Silene_swertiifolia	0	0	0	0	0	0	0.08	0	0	0	0	0
Stachys_lavandulifolia	0	0	0	0	0	0	0	0	0	0	0.5	0
Tulipa_micheliana	1	0	0	0	0	0	0.5	0	0	0	0	0
Verbascum_songaricum	0	0	0	0	0	0	0.5	0	0	0	0	0
Veronica_anagallisaquatica	0	0	0	0	0	0	0	0	0	0	0.5	0
Ziziphora_clinopodioides	0	0	0	0	0	0	0	0	0	0	0	0

WFS

Acantholimon_erinaceum	0	0	0	1	1	0	0	0	0	1	0	0	0
Acanthophyllum_crassinodum	0	0	1	0	0	0	1	0	0	0	0	0	0
Acanthophyllum_glandulosum	0	0	0	0	0	0	0	0	0	0	0	0	0
Allium_kuhsorkhense	0	0	2	0	0	0	0	0	0	0	0	0	0
Allium_tenuicaule	2	0	0	0	0	0	0	0	0	0	0	0	0
Alyssum_desertorum	0	0	0	0	0	0	0	0	0	0	0	0	0
Alyssum_stapfii	3	0	0	0	0	0	0	0	0	0	0	0	0
Arrhenatherum_kotschyi	0	0	3	0	0	0	0	0	0	0	0	0	0
Asperula_glomerata	0	0	0	0	0	0	0	0	0	0	0	0	0
Astragalus_citrinus	0	0	0	0	0	0	0	0	0	0	0	0	0
Astragalus_masanderanus	1	0	0	0	0	0	0	0	0	0	0	0	3
Astragalus_verus	0	0	0	0	0	0	0	0	0	0	1	0	0
Biebersteinia_multifida	0	0	0	0	0	0	0	0	0	0	0	0	0
Boissiera_squarrosa	0	0	2	0	0	1	0	0	0	0	0	0	0
Bromus_danthoniae	0	0	0	4	1	6	4	0	0	0	0	0	0
Bromus_oxyodon	0	0	6	0	0	0	0	2	0	0	0	0	0
Bromus_tectorum	0	4	0	0	0	0	0	0	0	0	0	0	0
Callipeltis_cucullaria	0	0	0	0	0	0	0	0	4	0	0	0	0
Centaurea_virgata	0	1	0	0	0	0	0	0	0	1	0	0	0
Cerastium_inflatum	0	0	0	0	0	0	3	0	1	0	0	0	0
Ceratocarpus_arenarius	0	0	0	0	0	0	0	0	0	0	0	0	0
Ceratocephala_falcata	0	3	0	0	0	0	0	0	5	0	0	0	0
Ceratocephala_testiculata	0	0	0	0	0	0	0	0	7	0	0	0	0
Cirsium_congestum	0	0	0	0	0	0	0	0	0	0	0	0	0
Cirsium_turkestanicum	0	0	0	0	0	0	0	0	0	0	0	0	0
Colchicum_robustum	0	0	0	0	0	0	0	0	0	0	0	0	0
Cousinia_concolor	0	0	2	2	2	0	0	0	0	0	0	0	0
Cousinia_multiloba	0	0	0	0	0	0	0	4	0	0	0	0	0
Cymbolaena_griffithii	0	0	0	0	0	0	0	0	6	0	0	0	0
Drabopsis_verna	0	0	0	0	0	0	0	0	2	0	0	0	0
Elwendia_afghanica	0	0	0	0	0	0	0	0	0	0	0	0	0
Elymus_hispidus	0	0	0	0	0	0	0	0	0	0	2	1	1
Eranthis_longistipitata	0	0	0	2	0	0	0	0	0	4	0	0	0
Eremurus_spectabilis	0	1	0	0	0	3	2	0	1	0	0	0	0
Eremurus_stenophyllus	0	1	0	0	3	0	0	1	0	0	0	0	1
Euphorbia_microsciadia	0	0	0	0	0	0	0	0	0	0	0	0	0
Euphorbia_szovitsii	0	0	0	0	0	0	0	0	0	0	2	2	0
Fallopia_convolvulus	0	0	0	0	0	0	0	0	0	2	0	0	0
Gagea_chomutowae	0	0	0	0	0	0	0	0	0	0	0	4	0
Gagea_gageoides	7	0	0	6	0	0	0	0	0	0	0	0	0
Gagea_kunawurensis	0	0	0	6	0	0	0	0	0	0	0	0	3
Gagea_reticulata	0	4	0	0	0	0	0	0	0	0	0	0	0
Gagea_setifolia	0	0	9	0	1	0	4	0	0	6	0	0	0
Galium_aparine	0	0	0	8	0	0	0	3	0	0	0	2	0
Galium_humifusum	0	0	0	0	0	0	0	0	0	0	0	0	7
Galium_tricornutum	5	0	8	0	8	0	0	0	0	6	3	0	0
Holosteum_glutinosum	0	0	0	0	0	1	2	0	0	0	0	0	0
Hypericum_scabrum	0	0	0	0	0	0	0	0	0	0	0	0	0
Ixiolirion_tataricum	1	0	0	0	0	1	0	0	0	0	0	0	0
Lactuca_orientalis	0	0	0	0	0	0	0	0	0	0	0	0	3
Lactuca_serriola	0	1	0	0	0	0	0	3	0	5	0	0	0
Lappula_sinaica	0	0	0	0	0	0	0	0	1	0	0	0	0
Lepyrodiclis_stellarioides	0	0	0	0	0	0	0	0	0	0	0	0	0
Melica_persica	0	0	0	0	0	0	0	0	0	0	0	0	0
Nepeta_bracteata	0	0	0	0	0	0	0	0	0	1	0	0	0
Noaea_mucronata	4	0	0	0	0	3	4	0	0	0	0	0	0
Onosma_dichroantha	0	0	0	0	0	0	0	0	0	1	0	0	1
Onosma_longiloba	0	0	0	0	0	0	0	0	0	0	0	0	2
Papaver_dubium	0	0	0	0	0	0	0	0	0	0	0	0	0
Perovskia_abrotanoides	0	0	0	0	0	0	0	0	3	0	3	0	0
Pimpinella_affinis	0	0	0	0	0	0	0	0	0	0	0	0	0
Poa_bulbosa	0	2	0	0	0	0	0	0	0	0	0	0	0
Poa_pratensis	0	0	5	0	0	0	0	0	0	0	0	0	0
Polygonum_aviculare	0	0	0	0	0	0	0	0	0	0	0	0	0
Polygonum_polycnemoides	0	0	0	0	0	0	0	0	0	0	0	0	0
Polygonum_thymifolium	0	0	0	0	0	0	0	0	0	0	0	0	0
Rheum_khorasanicum	0	0	0	0	0	0	0	0	6	0	0	0	0
Rumex_tianschanicus	1	2	0	1	2	2	0	0	0	0	0	1	0
Salvia_chloroleuca	0	0	0	0	0	0	0	0	0	0	0	0	0
Scandix_stellata	0	0	0	0	0	0	0	0	0	0	0	0	0
Scrophularia_crassipedunculata	0	0	0	0	0	0	3	0	0	0	0	0	0
Scrophularia_variegata	0	0	0	0	0	0	0	0	0	0	0	0	3
Silene_latifolia	0	2	0	0	0	0	0	0	0	0	0	0	0
Silene_swertiifolia	0	0	0	0	0	0	0	0	0	0	0	0	0
Stachys_lavandulifolia	0	0	1	0	0	0	0	0	0	0	2	0	0
Tulipa_micheliana	2	0	1	0	0	0	0	0	0	0	0	0	0
Verbascum_songaricum	0	0	0	0	0	0	0	0	0	0	0	0	0
Veronica_anagallisaquatica	2	0	0	0	2	0	0	0	0	0	0	0	0
Ziziphora_clinopodioides	0	0	0	0	0	0	1	0	0	0	1	0	0

Phlomis cancellata
Fereizi

CFS

Acantholimon sp. 1	4	0	0	1	0	0	3	0	0	0	0	3	1	3	0	0	0	0	0	1	4	2	1	3	2	3
Acantholimon sp. 2	0	0	0	0	0	0	1	0	0	0	0	0	0	1	0	0	0	0	0	0	0	1	0	0	0	0
Aethionema carneum	0	0	0	0	0	0	0	0	0	0	0	0	0	0	0	0	0	0	0	1	0	0	0	0	0	0
Aethionema trinervium var. apterocarpum	0	0	0	1	0	0	0	0	0	0	0	0	0	0	0	0	0	0	0	0	0	0	0	0	0	0
Alyssum stapfii	10	8	10	90	0	20	0	3	0	0	0	0	0	0	10	45	0	0	0	1	0	0	20	3	5	0
Amygdalus spinosissima subsp. Turcomanica	1	0	0	0	0	0	0	0	0	0	0	1	0	0	5	4	8	0	0	0	0	0	0	6	0	1
Androsace maxima	0	0	0	50	10	0	0	0	0	0	0	0	0	0	0	8	8	0	0	0	0	0	0	0	0	0
Artemisia khorassanica	0	0	0	0	0	0	6	0	0	0	0	0	1	2	0	0	17	8	0	10	17	9	0	0	0	0
Artemisia kopetdaghensis	10	16	22	10	8	5	0	16	4	17	7	0	0	0	0	0	0	0	0	0	0	0	0	0	0	4
Asperula glumerata	0	0	0	0	0	0	0	0	0	0	0	0	0	0	0	0	0	1	0	0	0	0	0	0	0	0
Astragalus chrysostachys	0	0	0	0	0	0	0	0	0	7	0	0	0	0	0	0	0	0	0	0	0	0	0	0	0	0
Astragalus fuhsii	0	0	0	0	0	0	0	0	0	0	3	0	1	0	0	0	0	1	0	0	0	0	0	0	0	0
Astragalus meschedensis	7	7	4	23	12	6	0	8	4	0	0	0	0	7	0	22	7	0	0	3	0	0	0	0	0	1
Astragalus oxyglottis	0	1	0	0	0	0	0	0	0	0	0	0	0	0	0	0	0	0	0	0	0	0	0	0	0	0
Biebersteinia multifida	0	0	0	0	0	0	0	0	0	0	0	0	0	0	0	0	0	0	0	0	0	0	0	0	1	0
Boissiera squarrosa	0	240	30	25	0	35	0	180	11	0	0	0	0	0	0	0	0	0	0	0	0	0	0	0	0	0
Bonium cylindricum	0	0	0	1	0	0	0	0	0	0	0	0	0	0	0	0	0	0	0	0	0	0	0	0	0	0
Bromus danthoniae	65	90	230	110	180	65	0	30	100	30	75	0	10	70	75	140	65	30	0	9	5	33	60	150	45	0
Bromus kopetdaghensis	0	0	0	0	0	0	0	0	0	6	0	0	1	0	0	0	0	0	0	0	0	0	0	0	0	0
Bromus oxyodon	0	0	0	0	0	0	0	0	0	0	0	0	0	3	0	0	0	0	0	2	0	0	0	0	0	0
Bromus tectorum	0	0	10	50	30	260	0	35	20	0	0	0	0	0	150	90	50	55	0	1	0	0	16	45	23	0
Buhsea trinervia	0	0	0	0	0	2	0	0	0	0	0	0	0	0	0	0	0	0	0	0	0	0	0	0	0	0
Bupleurum exaltatum	0	0	0	0	0	0	0	0	0	0	0	0	1	2	0	0	0	1	0	0	1	0	0	0	0	0
Callipeltis cucullaria	0	9	100	0	22	7	0	0	0	0	0	0	0	0	0	0	0	0	0	0	0	0	0	10	18	0
Centaurea behen	0	0	0	0	0	0	0	0	0	0	0	5	0	0	19	0	0	1	0	0	0	0	0	0	0	7
Centaurea virgata	0	11	2	0	3	4	0	0	1	0	3	0	0	0	7	11	8	0	0	0	0	0	1	10	0	0
Cerasus microcarpa	0	0	0	0	0	0	0	0	0	4	0	0	0	1	0	0	0	0	2	0	0	0	0	0	1	1
Cerasus pseudoprostrata	0	0	0	0	0	0	2	0	0	0	0	1	4	3	0	0	0	4	2	1	5	2	0	0	11	5
Chardinia orientalis	0	10	10	50	0	13	0	0	0	26	0	0	0	0	0	0	0	0	0	3	0	0	0	0	0	0
Chondrilla juncea	0	0	0	0	0	0	0	0	0	0	0	0	0	0	0	1	1	0	0	0	0	0	0	0	0	0
Colutea buhsei	0	0	0	0	0	0	0	0	0	0	0	0	0	0	0	0	0	0	0	0	2	0	0	0	0	1
Conringia perfoliata	2	0	0	0	0	0	1	0	0	0	0	0	0	0	0	0	0	0	0	0	0	0	0	0	0	0
Convolvulus lineatus	0	0	80	0	0	0	0	0	0	0	1	0	0	0	0	0	0	0	0	2	0	0	0	0	0	0
Cotoneaster nummularius	0	0	0	0	0	0	0	0	0	0	0	0	4	1	0	0	0	0	0	0	5	1	0	0	0	0
Cousinia freynii	7	7	0	0	3	0	0	2	3	0	0	0	0	0	0	0	0	0	0	0	0	0	0	0	0	0
Cousinia sp.	0	0	0	0	0	0	0	0	5	0	8	0	0	0	0	2	0	1	0	0	0	0	0	0	0	0
Cousinia termei	0	0	0	0	0	0	0	0	0	0	0	0	0	0	0	0	0	0	0	0	0	0	0	8	0	0
Crambe kotschyana	0	0	0	0	0	0	0	0	0	0	0	0	0	0	0	0	0	0	0	0	0	0	0	0	2	0
Crepis sancta	4	0	0	0	0	1	0	0	0	0	0	0	0	0	0	0	0	0	0	0	0	0	0	0	0	0
Crucianella gilanica	4	6	3	0	0	0	0	0	0	2	0	0	0	0	0	0	0	1	0	0	0	2	0	0	0	0
Cymbolaena griffithii	0	0	11	0	0	0	0	0	0	0	0	0	0	0	0	0	0	0	0	0	0	0	0	0	0	0
Dianthus crinitus	0	0	0	0	0	0	0	0	0	0	0	0	0	0	0	0	0	0	0	1	0	0	0	0	0	0
Dianthus orientalis	0	0	0	1	0	0	0	0	0	0	4	0	0	0	0	0	0	0	0	0	0	1	0	0	0	0
Dianthus sp.	0	0	0	0	0	0	0	0	0	2	0	0	0	0	0	0	0	0	0	0	0	0	0	0	0	0
Diathron vesiculosum	0	14	10	0	0	1	0	0	0	0	0	0	0	0	0	0	0	0	0	0	0	0	0	0	0	0
Echinops sp.	0	0	0	0	0	0	0	0	0	0	1	1	0	0	0	0	0	0	0	0	0	0	0	1	0	0
Elymus hispidus	0	0	0	0	0	0	0	0	0	0	35	0	0	0	0	0	0	0	0	0	0	20	0	0	0	0
Elymus transhyrcanus	0	0	0	0	0	0	7	0	0	3	0	9	10	6	0	0	0	3	15	6	24	0	15	2	33	28
Ephedra intermedia	0	0	0	0	0	0	0	0	0	0	0	0	0	4	0	0	0	0	7	0	0	0	0	0	0	1
Ephedra major	0	0	0	0	0	0	0	0	0	0	0	0	0	0	0	0	0	0	0	0	7	1	20	15	10	0
Ephedra sp.	0	0	0	0	0	0	0	0	0	0	0	0	0	0	0	0	0	1	0	0	0	0	0	0	0	0
Eremostachys labiosformis	0	0	0	0	0	0	0	0	0	0	0	0	0	0	0	0	1	0	0	0	0	0	0	0	0	0
Eremurus luteus	0	0	0	0	0	0	13	0	0	0	0	0	0	0	0	0	0	0	0	0	0	0	0	0	0	0
Eryngium bungei	2	5	0	2	0	3	0	0	5	4	4	0	1	2	0	1	0	1	9	2	0	3	4	0	0	11
Ferula ovina	0	0	0	0	0	0	6	0	0	0	0	1	0	0	0	0	0	0	6	0	0	0	0	0	0	0
Galagania plathypoda	0	0	0	2	0	0	0	0	0	0	0	0	0	0	0	0	0	0	0	0	0	0	0	0	0	0
Galium humifusum	0	0	6	0	0	0	0	0	0	0	0	0	0	0	0	0	0	0	0	0	0	0	0	0	70	20
Galium tricornutum	0	0	0	0	0	0	0	0	0	0	0	0	6	13	0	0	0	0	0	0	0	0	0	0	0	0
Glaucium elegans	0	0	0	0	0	0	0	0	0	0	0	0	0	0	0	0	0	0	0	0	0	0	0	1	0	0
Gundelia tournefortii	1	0	0	0	0	1	0	0	4	0	0	0	0	0	4	2	1	0	0	0	0	0	0	11	0	6
Haplophyllum acutifolium	0	0	0	0	0	0	0	0	0	0	0	1	4	1	1	3	0	10	0	0	0	0	1	0	0	1
Heteranthelium piliferum	0	90	75	2	30	0	0	0	0	0	0	0	0	0	0	0	0	0	0	0	0	0	0	0	0	0
Hymenocrater sessilifolius	0	0	0	0	0	0	4	0	0	0	0	0	0	0	0	0	0	3	11	0	0	0	0	0	4	0
Hypreicum scabrum	0	0	0	0	0	0	0	0	0	0	0	1	0	1	0	0	0	0	2	0	0	0	0	0	0	1
Ixiolirion tataricum	0	0	0	0	0	0	1	0	0	0	0	0	0	0	0	0	0	0	0	0	0	0	0	0	0	0
Koelpinia linearis	0	0	8	0	0	0	0	0	0	0	0	0	0	0	0	0	0	0	0	0	0	0	0	0	0	0
Lactuca serriola	0	3	13	0	0	5	1	2	2	0	2	0	0	0	0	0	0	0	0	0	5	1	3	0	2	1
Lappula microcarpa	0	1	1	0	0	0	0	0	0	0	2	0	0	0	0	0	0	0	0	0	0	0	0	0	0	0
Lonicera nummulariifolia	0	0	0	0	0	0	0	0	0	0	0	0	1	0	0	0	0	0	1	0	4	0	0	0	0	2
Medicago sp.	0	5	0	0	0	0	0	0	0	0	0	0	0	0	0	0	0	0	0	0	0	0	0	0	0	0
Melica persica	0	0	0	0	0	0	0	0	0	0	0	0	0	0	0	0	0	0	0	0	0	1	0	1	0	0
Meristotropis xanthoides	0	0	0	0	0	0	0	0	0	0	2	0	0	0	0	0	0	0	0	0	0	0	0	0	0	0
Minuartia meyeri	0	0	0	0	10	50	0	0	0	0	0	0	0	8	0	0	0	0	0	0	0	0	0	0	0	0
Muscari neglectum	0	0	0	0	0	0	0	0	0	0	0	0	0	0	0	0	0	0	1	0	0	0	0	0	0	0
Nigella integrifolia	0	0	0	0	0	0	0	0	0	4	0	0	0	0	0	0	0	0	0	0	0	0	0	0	0	0
Onobrychis khorassanica	0	0	0	0	0	0	0	0	0	0	0	0	0	0	0	0	0	0	0	0	0	0	0	0	0	3
Papaver dubium	0	0	0	0	0	0	0	0	0	0	0	0	0	0	0	0	0	0	0	0	0	0	0	0	2	0
Perovskia abrotanoides	0	0	0	0	0	0	0	0	0	0	0	0	0	0	0	0	0	0	9	0	0	0	0	0	0	0
Poa bulbosa	0	0	0	0	0	0	0	0	0	15	46	0	200	150	0	30	0	15	0	23	0	18	40	0	20	0
Polygonum paronychioides	0	0	0	5	11	1	0	2	1	0	0	0	0	0	0	0	0	0	0	0	0	0	0	15	0	0
Prangos latilobia	0	0	0	0	0	0	0	0	0	0	0	0	0	0	0	1	2	0	0	0	0	0	0	0	0	0
Rochelia cardiosepala	0	0	0	0	2	0	0	0	0	0	0	0	0	0	0	0	0	0	0	0	0	0	1	0	0	0
Roemeria hybrida	0	0	0	0	0	0	2	0	0	0	0	0	0	0	0	0	0	0	0	0	0	0	0	0	3	0
Rosa persica	0	0	0	0	0	0	0	1	5	0	0	0	0	1	80	0	0	12	0	0	0	1	0	0	0	0
Rubia florida	0	0	0	0	0	0	0	0	0	0	0	0	0	0	1	0	0	0	0	0	0	0	0	0	0	1
Salvia ceratophylla	0	0	0	0	0	0	0	0	0	0	0	0	0	0	0	0	0	0	0	0	0	0	1	3	1	0
Salvia chloroleuca	0	0	0	0	0	0	2	0	0	0	0	4	0	0	0	2	0	0	4	0	0	0	0	0	0	12
Sanguisorba minor	0	1	0	0	0	0	0	0	0	0	0	0	0	0	0	0	0	0	0	0	0	0	0	0	0	0
Scabiosa rotata	0	2	0	0	0	1	0	0	0	0	0	0	0	0	0	0	0	0	0	0	0	0	0	0	0	0
Scandix stellata	60	0	5	3	5	0	170	0	0	0	0	0	5	20	0	65	0	0	0	0	0	8	0	35	28	0
Scariola orientalis	0	0	0	0	0	0	0	0	0	0	0	0	1	0	2	1	0	0	0	0	0	0	0	0	0	0
Serratula latifolia	0	0	0	0	0	0	0	0	0	0	0	0	35	35	0	0	0	20	8	25	0	0	35	18	12	30
Silene bruhaica	0	0	1	0	0	0	0	0	0	0	0	0	0	0	0	0	0	0	0	0	0	0	0	0	0	0
Silene coniflora	0	0	0	0	0	0	0	0	0	0	0	0	0	0	0	0	0	0	0	1	0	0	0	0	4	4
Silene swertiifolia	0	0	0	0	0	0	0	0	0	0	0	0	0	0	0	0	0	0	0	0	0	0	0	2	0	0
Sonchus oleraceus	0	0	0	0	0	0	0	0	0	0	0	0	0	1	0	0	0	0	0	0	0	0	0	0	0	0
Stachys lavandulifolia	0	0	0	0	0	0	0	7	5	0	0	0	0	1	0	0	0	0	0	0	0	0	0	0	0	0
Stipa barbata	0	0	0	0	0	0	0	0	0	0	0	0	0	0	0	1	0	0	0	2	0	0	0	0	0	0
Stipa hohenackeriana	0	1	15	6	10	1	0	0	0	3	0	0	0	0	0	0	0	0	0	0	0	0	0	0	0	0
Stroganowia affghana	2	0	0	0	0	0	0	0	0	2	2	3	0	0	0	0	0	0	0	0	0	0	0	0	0	0
Taeniatherum caput-medusae	0	450	300	170	185	120	0	100	80	0	0	0	0	0	48	90	140	0	0	17	0	10	20	180	0	0
Thlaspi perfoliatum	0	0	0	0	0	0	0	0	0	0	0	0	15	0	0	0	0	0	0	0	0	0	0	0	0	0
Trichodesma bamianicum	0	1	0	0	0	0	0	0	0	0	1	0	0	0	0	0	0	0	0	0	0	0	0	0	0	0
Trigonella monantha	0	0	0	0	30	0	0	0	0	0	0	0	0	0	0	0	0	0	0	0	0	0	0	0	0	0
Turgenia latifolia	0	0	0	1	0	0	0	0	0	0	0	0	0	0	0	0	0	0	0	0	0	0	0	0	0	0
Valerianella tuberculata	0	0	2	0	0	0	0	0	0	0	0	0	0	0	0	0	0	0	0	0	0	0	0	0	0	0
Verbascum cheiranthifolium	0	3	0	0	0	1	0	0	2	0	2	4	0	0	1	2	1	0	2	0	8	0	2	2	0	0
Veronica sp.	20	0	0	0	0	0	60	0	0	0	0	0	0	0	0	0	0	0	0	0	0	2	0	0	0	15
Ziziphora clinopodioides	0	0	0	0	0	0	0	0	0	0	0	0	0	0	0	0	0	0	0	0	3	0	0	0	0	0
Ziziphora tenuior	4	20	45	0	0	5	0	0	0	0	0	0	0	0	130	10	15	0	0	0	0	0	0	11	9	0

WFS

Acantholimon sp. 1	4	0	1	1	0	1	6	0	8	0	2	0	0	0	4	0	0	0	0	0	4	2	1	17	0
Acantholimon sp. 2	0	0	0	0	0	0	0	0	0	0	1	0	0	0	0	0	0	0	0	0	0	0	0	0	0
Acanthophyllum mucronatum	0	0	0	0	0	0	0	0	0	0	0	0	0	0	0	0	0	0	0	0	1	0	0	0	0
Allium oschaninii	0	0	0	0	0	0	0	0	0	0	0	0	0	23	0	0	0	0	0	0	0	0	0	0	0
Allium rubellum	0	0	0	0	0	0	0	0	3	0	0	0	0	0	0	0	0	0	0	0	0	0	0	0	0
Allium sp.	0	0	0	0	0	1	0	0	0	0	0	0	0	0	0	0	0	0	0	0	0	0	0	0	0
Alyssum stapfii	0	35	10	3	0	0	0	0	0	6	0	0	0	0	1	0	22	0	0	8	0	0	3	0	10
Amygdalus spinosissima subsp. Turcomanica	1	0	0	0	0	0	0	0	0	0	0	0	0	2	4	0	0	0	0	0	0	0	0	0	0
Androsace maxima	0	0	0	0	0	0	0	0	0	6	1	0	0	0	0	0	0	0	0	0	0	0	0	0	0
Arrhenatherum kotschyi	0	0	0	0	1	0	0	0	0	0	0	0	0	0	0	0	0	0	0	0	0	0	0	0	0
Artemisia khorassanica	0	0	0	1	2	0	0	0	0	0	0	0	0	0	0	15	0	0	13	6	3	0	25	18	0
Artemisia kopetdaghensis	15	14	20	0	0	0	0	18	11	12	10	0	0	0	0	0	0	0	0	0	0	0	0	0	13
Asperula glumerata	1	0	0	0	3	0	0	0	0	0	0	0	0	0	1	2	0	0	0	0	0	0	0	1	0
Astragalus ackerbergensis	0	0	0	0	0	0	0	0	0	0	0	0	0	0	0	4	0	0	0	0	0	0	0	0	0
Astragalus anacamptus	0	0	7	0	0	0	0	0	0	0	0	0	0	0	0	0	0	0	0	0	0	0	0	0	0
Astragalus dipelta	5	0	0	0	0	0	0	0	0	0	0	0	0	0	0	0	0	0	0	0	0	0	0	0	0
Astragalus meschedensis	4	5	8	5	2	4	0	12	4	6	14	0	0	0	5	1	0	0	0	1	4	0	0	5	0
Boissiera squarrosa	0	0	5	0	0	0	0	75	0	0	8	0	0	0	0	0	0	0	0	0	0	5	0	0	0
Bromus danthoniae	5	90	120	3	2	0	0	9	100	33	70	0	0	0	45	65	250	0	0	35	9	21	40	25	0
Bromus oxyodon	0	0	25	0	0	0	0	0	0	0	0	0	0	0	0	0	0	0	0	0	0	0	0	0	0
Bromus tectorum	0	40	45	27	0	45	0	30	0	80	20	0	0	0	110	0	110	25	0	0	0	75	75	0	0
Bupleurum exaltatum	0	0	0	0	0	0	0	0	0	0	0	0	0	0	0	0	0	0	0	0	3	0	0	2	0
Callipeltis cucullaria	0	0	0	0	0	0	0	0	0	0	0	0	0	0	1	0	0	0	0	0	0	0	0	0	0
Centaurea behen	0	0	0	0	0	0	0	0	0	0	0	0	4	0	0	0	73	0	0	0	0	0	0	0	0
Centaurea virgata	0	1	0	0	0	0	0	4	0	0	0	0	0	0	0	1	16	11	0	0	0	4	0	0	4
Cerasus microcarpa	0	0	0	0	0	0	0	0	0	0	0	0	0	0	0	0	0	0	1	0	0	0	0	0	0
Cerasus pseudoprostrata	0	0	0	0	0	1	0	0	0	6	0	0	0	0	0	0	0	0	0	0	0	0	0	0	1
Chardinia orientalis	0	0	0	0	0	0	0	0	0	0	3	0	0	0	0	0	0	0	0	0	0	0	0	0	15
Chenopodium botrys	0	0	0	0	0	0	0	0	0	0	0	0	0	0	0	0	0	0	0	0	0	0	0	0	1
Colutea buhsei	0	0	0	0	0	0	0	0	0	0	0	0	0	0	0	0	1	0	0	0	0	0	0	0	0
Conringia perfoliata	1	0	0	0	0	0	0	0	0	0	0	0	0	0	0	0	0	0	0	0	0	0	0	0	0
Convolvulus pseudocantabricus	0	0	0	0	0	0	0	0	0	0	0	0	0	0	0	0	0	0	0	0	0	1	0	0	0
Cousinia freynii	5	2	0	0	0	0	0	0	25	2	0	0	0	0	0	0	0	0	0	0	0	0	0	0	0
Cousinia sp.	0	0	0	0	0	0	0	0	2	0	1	0	1	0	0	0	0	0	0	0	0	0	0	8	0
Cousinia termei	0	0	0	0	1	3	0	0	0	0	0	0	0	0	11	0	0	0	0	0	1	0	0	0	0
Crepis sancta	0	0	0	0	0	0	0	0	0	0	1	0	0	0	0	0	0	0	0	0	0	0	0	0	0
Crucianella gilanica	1	0	0	0	0	0	0	0	4	0	6	0	0	0	0	0	0	0	0	0	0	0	0	0	0
Cymbolaena griffithii	0	0	0	0	0	0	0	0	0	0	2	0	0	0	0	0	0	0	0	0	0	0	0	0	0
Dianthus orientalis	0	0	0	0	0	0	0	0	0	0	1	1	0	0	0	0	0	0	0	0	0	0	0	0	0
Echinops sp.	0	0	0	0	0	0	0	0	0	0	0	0	0	0	0	0	1	0	1	0	0	0	0	0	0
Elymus transhyrcanus	0	0	0	3	3	6	0	0	1	0	0	4	3	0	0	0	0	0	11	49	8	0	1	1	0
Ephedra intermedia	0	0	0	0	0	0	0	0	0	0	0	0	0	0	0	9	0	0	0	0	0	0	0	0	0
Ephedra major	0	0	0	0	0	0	0	0	0	0	0	11	0	0	0	0	0	0	0	0	0	3	0	0	0
Eremopoa persica	0	0	0	0	0	0	5	0	0	0	0	0	0	0	0	0	0	0	0	0	0	0	0	0	0
Eremostachys labiosformis	0	0	0	0	0	0	0	0	0	0	0	0	0	1	0	0	0	0	0	0	0	0	0	0	0
Eryngium bungei	0	0	0	0	0	0	0	0	4	0	2	0	1	0	0	0	0	7	3	10	2	0	9	1	1
Euphrasia pectinata	0	0	0	1	0	0	0	0	0	0	0	0	0	0	0	0	0	0	0	0	0	0	0	0	0
Ferula flabelliloba	0	0	0	0	0	0	0	0	0	0	0	0	0	0	2	0	0	0	0	0	0	0	0	0	0
Ferula ovina	0	0	0	1	0	0	8	0	0	0	0	0	3	0	0	0	0	0	0	0	0	0	0	0	0
Festuca pratensis	0	0	0	0	0	0	0	0	0	0	0	0	0	0	0	2	0	0	0	0	0	0	0	0	0
Galium humifusum	0	0	0	3	1	3	0	0	0	0	12	0	0	0	0	0	0	0	0	0	0	0	0	0	5
Glaucium elegans	0	0	0	6	4	1	31	0	0	0	0	0	0	0	0	0	0	0	0	0	0	0	0	0	0
Gundelia tournefortii	1	0	0	0	0	0	0	0	0	0	0	1	0	0	0	0	5	4	0	0	0	4	0	0	4
Haplophyllum acutifolium	2	0	0	0	0	0	0	0	0	0	0	0	0	0	0	10	0	0	0	0	0	0	0	0	0
Hymenocrater sessilifolius	0	0	0	0	0	0	3	0	0	0	0	2	0	0	0	0	0	0	0	0	0	0	0	0	0
Hypreicum scabrum	0	0	0	0	0	0	0	0	0	0	0	0	0	0	0	0	0	0	0	0	0	0	0	0	1
Lactuca serriola	1	0	0	0	0	0	5	1	0	0	0	0	1	0	0	0	0	0	0	0	1	0	0	0	0
Lamium amplexicaule	0	0	0	15	0	2	0	0	0	0	0	0	0	0	0	0	0	0	0	0	0	0	0	0	0
Lappula microcarpa	0	0	0	0	3	0	1	0	0	0	0	0	0	0	0	0	0	0	0	0	0	0	0	0	0
Leontice leontopetalum	0	0	0	0	1	0	0	0	0	0	0	0	0	0	0	0	0	0	0	0	0	0	0	0	0
Melica persica	0	1	0	0	0	0	0	0	0	0	0	0	0	0	0	0	0	0	0	0	0	0	1	6	0
Minuartia meyeri	0	0	0	0	0	0	6	0	0	0	0	0	0	0	0	0	0	0	0	0	0	0	0	0	8
Nigella integrifolia	0	0	10	0	0	0	0	0	0	0	3	0	0	0	0	0	0	0	0	0	0	0	0	0	0
Noaea mucronata	0	0	0	0	0	0	0	0	0	0	0	0	0	0	0	3	0	0	0	0	0	0	0	0	0
Papaver dubium	0	0	0	0	0	0	0	0	0	0	0	0	0	0	0	0	0	0	0	0	0	1	0	0	0
Perovskia abrotanoides	0	0	0	0	0	0	0	0	0	0	0	16	20	0	0	0	0	7	6	0	0	0	0	0	65
Poa bulbosa	0	110	0	0	0	0	0	0	40	0	7	0	0	0	0	4	0	0	0	26	15	0	0	0	0
Polygonum paronychioides	2	2	3	5	0	0	0	1	0	1	3	0	0	0	0	0	0	0	0	0	0	0	2	0	0
Polygonum polycnemoides	0	0	0	0	0	0	0	0	0	0	0	0	0	0	1	0	0	0	0	0	0	0	0	0	0
Prangos latilobia	0	0	0	0	0	0	0	0	0	0	0	0	0	0	1	0	0	0	0	0	0	0	0	0	0
Rochelia cardiosepala	0	0	0	0	0	0	0	0	0	0	2	0	0	0	0	0	0	0	0	0	0	0	0	0	2
Rosa beggeriana	0	0	0	0	0	0	0	0	0	0	0	0	0	0	0	0	0	0	1	0	0	0	0	0	0
Rosa persica	5	13	0	0	0	0	0	0	5	0	0	0	0	0	1	1	0	0	0	0	0	0	0	0	0
Rubia florida	0	0	0	0	0	0	0	0	0	0	0	0	0	0	2	0	0	0	0	0	2	3	0	0	0
Scandix stellata	12	0	0	6	3	4	25	0	0	0	4	0	0	0	30	3	0	0	0	40	0	180	0	0	0
Scariola orientalis	0	0	0	0	0	0	0	0	0	0	0	0	0	0	1	0	1	0	0	0	0	0	0	0	0
Scutellaria litwinowii	0	0	0	0	0	0	0	0	0	0	0	0	0	0	1	0	0	0	0	0	0	0	0	0	0
Serratula latifolia	0	0	0	0	0	0	0	0	0	0	0	0	0	0	0	17	0	0	0	37	2	30	0	0	0
Silene coniflora	0	0	4	0	0	0	0	0	0	0	0	0	0	0	0	0	0	0	0	0	0	0	0	3	0
Silene noctiflora	0	0	0	0	0	0	0	0	0	0	0	0	0	0	3	0	0	0	0	0	0	0	0	0	0
Stachys lavandulifolia	0	20	20	0	0	0	0	0	0	6	0	0	0	0	0	0	0	0	0	0	0	0	0	0	0
Stipa barbata	0	0	0	0	0	0	0	0	0	0	0	0	0	0	0	0	0	0	0	0	0	0	5	0	0
Stipa hohenackeriana	2	3	0	0	0	0	0	0	0	0	15	0	0	0	0	0	0	0	0	0	0	0	0	0	0
Stroganowia affghana	2	0	5	0	0	1	0	0	3	0	0	0	0	0	0	0	0	0	0	0	0	0	0	0	0
Taeniatherum caput-medusae	0	50	30	0	0	0	0	83	0	98	27	0	0	0	0	40	200	55	0	0		250	20	0	0
Trichodesma incanum	0	0	0	0	0	0	0	0	0	0	0	0	0	0	0	0	1	0	0	0	0	0	0	0	0
Verbascum cheiranthifolium	0	0	0	1	0	0	0	0	0	0	0	0	3	4	0	0	1	3	2	3	0	0	0	0	1
Veronica sp.	0	20	15	150	1	0	0	0	0	1	3	0	0	0	0	0	0	0	0	5	3	0	8	0	0
Ziziphora clinopodioides	0	0	0	0	0	0	0	0	0	0	0	0	0	1	0	0	0	0	0	0	0	0	0	0	0
Ziziphora tenuior	10	0	0	0	0	0	0	0	0	0	16	0	0	0	0	0	0	0	0	0	0	10	0	0	0

Phlomis cancellata
Heydari

CFS

Acantholimon_erinaceum	2	4.5	12	0	11	10	6.5	1	0	0
Acantholimon_quinquelobum	0	0	0	0	0.1	0	0	0	0	0
Acantholimon_raddeanum	16.6	24	1	0	0	0	0.1	5	1	0
Acanthophyllum_glandulosum	0	0	0	0.5	1	0	0	0	2.5	0
Achillea_santolinoides	0	0.5	1	0	0	0	0	0	0	0
Alyssum_alyssoides	0	0	0	0	0	0	0	0	0	0
Alyssum_desertorum	0	0	0.1	0	0	0	0	0	0	0
Alyssum_heterotrichum	0	0	0	0	0	0	0	0	0	0
Alyssum_lanceolatum	0	0	0	0	0	0	0	1.5	0	0
Alyssum_linifolium	0	0	0	0	0	0	0.1	0	0	0
Alyssum_singarense	0	0	0	0	0	0	0	0	0	0
Alyssum_szovitsianum	0	0	0	0	0.5	0.7	0	0	0	0
Artemisia_kopetdaghensis	0	2.3	7	0	3	0	0	4	0	0
Asperula_setosa	0	0	0	0.2	0	0	0	0	0.5	0
Astracantha_cerasocrena	0	0	0	0	0	0	0.2	0	0	0
Astragalus_masenderanus	0	0	0	0	0	0	0	6	0.2	0
Astragalus_mercklinii	0	0	0	0	0	0	0	2	0	0
Astragalus_verus	7	12	3.7	0	19	1.5	0.2	0	0.2	0
Atraphaxis_spinosa	0	0	0	0	0	0	0	0	0	0
Berberis_integerrima	0	0	0	0.5	0	0	0	0	0	0
Boissiera_squarrosa	0	0	0	0	0	0.1	0	0	0	0
Bromus_danthoniae	0	0	0	0	1.5	0	0	0.1	0	0
Bromus_kopetdagensis	10	0	0	0	0	0	0	1	2	0.1
Bromus_tectorum	0	0	0	0	1.5	0	0	0	0	0
Bufonia_oliveriana	0	0	0	0	0	0	0	0	0	0
Bunium_afghanicum	0	0.5	0	0	0	0	0	0	0	0
Bunium_cylindricum	0	0	0	0	0	2	0	0	0	0
Bupleurum_falcatum	0	0	0	0	0	0	0	0	0	0
Callipeltis_cucullaris	0	0	0	0	0	0	0	0	0	0
Centaurea_virgata	0	0	0	0	0	0	0.4	0	0	0
Cerastium_dichotomum	0	0	0	0	0	0	0	0	0	0
Ceratocephala_falcata	0.5	0.5	3	0	0	0.1	0.1	0.1	0	0
Ceratocephala_testiculata	0	0	0	0	0	0	0	0	0.2	0
Chardinia_orientalis	0	0	0	0	0	1	0	0	0	0
Cirsium_bornmuelleri	0	5	0	0	0	0	0	0	3	0
Clinopodium_graveolens	0	0	0	0	1	0	0	0	0	0
Colchicum_robustum	0	0	0	0	0	0	0.2	0	0	0
Conringia_clavata	0	0	0	0	0	0.5	0	0	0	0
Convolvulus_arvensis	0	0	0	0	0	0	0	0	0	0
Convolvulus_lineatus	7	1.5	0	0	0	0	0	0	0	0
Cotoneaster_kotschyi	0	0	0	1	0	0	0	0	0	0
Cousinia_bienerti	0	0	0	0	0	0	0	0.2	3.5	0.4
Cousinia_eryngioides	0	0	0	0	0	0	0	0	0	0
Cousinia_freynii	0	0	0	0	0.3	0.7	0.2	0	0	0
Cousinia_lasiolepis	0	0	0	0	0	0	0	0	0	0.3
Cousinia_microcarpa	0	0	0.3	0	0	0	0	0	0	0
Cousinia_smirnowii	0	0	0	0	0	0	0	0	0.2	0
Crucianella_chlorostachys	0	0	0	0	0	0	0	0	0	0
Crucianella_gilanica	0	0	0	3	0	0	0	0	0.5	0
Cymbolaena_griffithii	0	0	0	0	0	0	0	0	0	0
Draba_nuda	0	0	0	0	0	0	0	0	0	0
Echinops_ritrodes	0	0	0	0	0	0	0	0.1	0	0
Elymus_hispidus	0	0	10	1.5	0	0	0	0	2.2	0
Eremopyrum_bonaepartis	0	0	0	0	0	1	0	0	0	0
Eremurus_spectabilis	0	0	6	0	0	0	0	0	0	0
Eremurus_stenophyllus	0	0	0	0	0	0	0	0.1	0	0
Eryngium_billardierei	0	0	0	0.5	0	0	0	0	0.3	1.5
Eryngium_bungei	0	0	0	0	0.1	0.5	0.1	0	0	0
Erysimum_ischnostylum	0	0	0	0	0.1	0	0	2	0	0.5
Euphorbia_boissieriana	0	0	0	0	0	0	0	0	0.5	0
Euphorbia_microsciadia	0	0	0	0	0	0	0	0	0.7	0
Ferula_gummosa	0	0	0	0	0	0	0	0	0.3	0
Festuca_valesiaca	0	0	1.6	0	0	0.2	0	0.2	0	0
Fumaria_vaillantii	0	1	0	0	0	0	0	0	0	0
Gagea_gageoides	0	0	0	0	0	0	0.1	0	0	0
Gagea_kunawurensis	0	0	0	0	0	0.1	0	0	0	0
Galium_spurium	0	0	0	0	0	0	0	0	0	0.1
Garhadiolus_hedypnois	0	0.5	0	0	0	0	0	0	0	0
Geranium_kotschyi	0	0	0	0	0	1	0	0	0	0
Gladiolus_atroviolaceus	0	0	0	0	0	4	0	0	0	0
Henrardia_persica	0	0	0	0	0.5	0.1	0	0	0	0
Heteranthelium_piliferum	0	0	0	0	0	0	0	0.1	0	0
Holosteum_umbellatum	0	0	0	0	0.1	0.5	0	0	0	0
Hymenocrater_platystegius	0	0	0	0.5	0	0	0	0	0	0
Hypecoum_pendulum	0	0	0	0	0	1.5	0	0	0	0
Iris_fosteriana	0	0	0	0	0	0	0.7	1	0	0
Ixiolirion_tataricum	0	0	0	0	0	0	0	0	0	1
Johrenia_platycarpa	0	0	0	0	0	0	0	0	0	0
Jurinea_sintenisii	0	0	0	0	0	0	0	0	1.2	0
Krascheninnikovia_ceratoides	0	0	0	0	0	0	0	7.5	0	0
Lactuca_orientalis	2	0	0	0.3	0	0.2	1	0	0	0.3
Lagochilus_cabulicus	0	0	0	0	0	0	0.2	0	0	0
Lappula_barbata	0	0	0	0	0	0	0	4	0	0
Lappula_microcarpa	0	0	0	0	0	0	0	0	0	0
Leymus_secalinus	0	0	0	0	0	0	0	0	1.5	7.5
Loliolum_subulatum	0	0	0	0	0	0	0	0	0	0
Lotus_corniculatus	0	0	0	3	0	0	0	0	0	0
Melica_persica	0	0	0	0	0	0	0	0	0	0
Minuartia_meyeri	0	0	0	0	2	0	0	0	0	0
Myosotis_stricta	0	0	0	0	0	0	0	0	0	0
Noaea_mucronata	0	0	0	0	0.2	0	0	0.1	0	0
Onobrychis_cornuta	0	0	0	0	0	0	0	0	0	0
Onobrychis_verae	0	0	0	0	0	0	0.1	9	0	0
Perovskia_abrotanoides	0	0	0	0	0	0	0	0	0	0
Phlomoides_binaludensis	0	0	0	0	0	0.1	0	0	0	0
Poa_bulbosa	5	8	8	1	2	0	0	0	0	0
Polygonum_thymifolium	0	0	0	0	0	0	0	0	3.5	0
Prunus_microcarpa	0	0	0	0.5	0	0	0	0	0	0
Prunus_pseudoprostrata	0	0	0	0	0	0	0	0	0.1	0
Rhaponticum_repens	0	5	0	0	0	0	0	0	0	0
Rhizocephalus_orientalis	0	0	0	0	2	0	0.1	0	0	0.1
Rochelia_disperma	0	0	1	0	0.1	0	0	0	0.2	0
Rochelia_peduncularis	0	0.5	0	0	0	0	0	0	0.3	0
Rochelia_persica	0	0	0	0	0	0	0	0	0	0
Salvia_chloroleuca	0	0	0	0	0.2	0	0	0	0	0
Sanguisorba_minor	0	0	0	0	0	0	0	0	0	0
Scandix_stellata	0	0	0	0.1	0	0	0	0	0	0
Scorzonera_leptophylla	0	0	1	0	0	0	0	0	0	0
Scutellaria_luteocoerulea	0	0	0	0	0	0	0	0	0	0
Senecio_paulsenii	0	0	0	0	0	0	0	0.3	0	1.7
Serratula_latifolia	0	0	0	9	0	0	8	7	0	8
Silene_bupleuroides	0	0	0	0	0	0	0	0	4	0
Silene_indeprensa	0	0	0	0	0	0	0	0	0	0
Stachys_lavandulifolia	0	0	0	0	0	0.5	0	0	0	0
Stellaria_alsinoides	0	0.5	0	0	0	0	0	0	0	0
Stipa_hohenackeriana	0	0	0	0	0	1	0	1	0	0
Stipa_holosericea	0	0	0	0	3	0	1	0	0	0
Stipa_lessingiana	0	0	0.5	0	0	0	0	0	0	0
Taeniatherum_caputmedusae	0	0	0	0	0	10	0	0	0	0
Taraxacum_afghanicum	0	0	0	0	0	0	0	0	0	0
Thalictrum_isopyroides	0	0	0	12	0	0	0	0	0	2
Tulipa_micheliana	0	0	0	0	0	0	0	0	0	1
Verbascum_cheiranthifolium	0	0	0.5	0	1	1	2	0	0.2	0
Veronica_arguteserrata	0	1	2	0	0	0	0	0	0	0
Veronica_campylopoda	1	0	0	1	0	0	0	0.5	0	0
Veronica_rubrifolia	0	0	0	0	0	0	0	0	0	0.1
Vicia_subvillosa	0	0	0	0	0	0	0	1	0	1
Viola_occulta	0	0	0	0	0.1	0.5	0	0	0	0

WFS

Acantholimon_erinaceum	0	3.8	0	14	40	11	8.5	0.1	9.5	2.2
Acantholimon_quinquelobum	0	0	0	0	0	0	0	0	0	0
Acantholimon_raddeanum	24	0	0	0	0	1	0	2	0.2	0
Acanthophyllum_glandulosum	0	0	0	7.5	0	10	3	2	3.6	3
Achillea_santolinoides	0	0	0	0.1	0	0	0	0.2	0.5	0
Alyssum_alyssoides	0	0	0	0.5	0	0	0	0	0	0
Alyssum_desertorum	0	0	0	0	0.1	0	0	0	0	0
Alyssum_heterotrichum	0	0.5	0	0	0	0	0	0	0	0
Alyssum_lanceolatum	0	0	0	0	0	0	0	0	0	0
Alyssum_linifolium	0	0	0	0	0	0	0	0	0	0
Alyssum_singarense	0	1	0	0	0	0	0	0	0	0
Alyssum_szovitsianum	0.5	0	0	0	0	0	0	0	0	0
Artemisia_kopetdaghensis	0	0	0	4.5	0	0	0.2	0	0	1
Asperula_setosa	0	0	0	0	0	0	0	0.1	0	0
Astracantha_cerasocrena	0	0	0	0	0	0	0	0	0	0
Astragalus_masenderanus	0	0	0	0	0	0	0	0	0	0
Astragalus_mercklinii	0	0	0	0	0	0	0	0	0	0
Astragalus_verus	12	20	0.5	13	2	0.2	5	3	4.2	1.7
Atraphaxis_spinosa	0	0	0.3	0	0	0	0	0	0	0
Berberis_integerrima	0	0	0	0	0	0	0	0	0	0
Boissiera_squarrosa	0	0	0	1	0.1	0.1	0	0	0	0
Bromus_danthoniae	0	0	0	0.1	0.2	1	0	0.1	0	0
Bromus_kopetdagensis	0	0	0	3	0	0	0	0	0	0
Bromus_tectorum	0	0	0	0.1	0	0.1	1	0	0	0
Bufonia_oliveriana	0	0	0	0	0	0.1	0	0	0	0
Bunium_afghanicum	0	0	0	0	0	0	0.5	0	0	0
Bunium_cylindricum	0	0	0	1	0	0	0	0	0	0
Bupleurum_falcatum	0	0	0	0	0	0.5	0	1	12	5
Callipeltis_cucullaris	0	0.1	0	0	0	0	0	0	0	0.1
Centaurea_virgata	0	0	7	0	0	0	0	0.5	7	0
Cerastium_dichotomum	0	0	0	0	0	0	0.1	0	0	0
Ceratocephala_falcata	0.5	0.5	0	0.1	0	0.2	0	0	0.1	0
Ceratocephala_testiculata	0	0	0	0	0	0	0	0	0	0
Chardinia_orientalis	0	0	0	0	0	0	0	0	0	0
Cirsium_bornmuelleri	1	0.7	0.2	0	0	0	0	0	0	0.1
Clinopodium_graveolens	0	0	0	0	0	0	0	0	0	0
Colchicum_robustum	0	0	0	0	0	0	0	0	0	0
Conringia_clavata	0	0	0	0	0	0	0	0	0	0
Convolvulus_arvensis	0	0	0	0	1	0	0	0	2.5	0.1
Convolvulus_lineatus	0	0	0	0	0	0	0	0	0	0
Cotoneaster_kotschyi	0	0	0	0	0	0	0	0	0	3.3
Cousinia_bienerti	0	0	0	0	0	0.3	0	0	0	0
Cousinia_eryngioides	0	0	0	0	7	0	0	0	0	0
Cousinia_freynii	1	0	0	0.7	0	0	0	0	0.5	6
Cousinia_lasiolepis	0	0	0	0	0	0	0	0	0	0
Cousinia_microcarpa	0	0	0	0	0	0	0	0	0	0
Cousinia_smirnowii	0	0	0	0	0.3	0.1	0	0	0	0
Crucianella_chlorostachys	0	0.2	0	0	0	0	0	0	0	0
Crucianella_gilanica	0	0	0	0	0	0	0.2	0	0.4	0
Cymbolaena_griffithii	0	0	0	0	0	0	0.1	0	0	0
Draba_nuda	0	0	0	0	0	0	0.1	0	0	0
Echinops_ritrodes	0	0	0	0	0	0	0	0	0	0.3
Elymus_hispidus	10	2	0	0	0	0	0	30	0	0.5
Eremopyrum_bonaepartis	0	0	0	0	0	0	0	0	0	0
Eremurus_spectabilis	0.1	7.5	0	1	0	0	0.5	0	0	0
Eremurus_stenophyllus	0	0	0	0	0	0	0	0	0	0
Eryngium_billardierei	0.5	0	0	0	0	0	0	0	0	0
Eryngium_bungei	0	0	0	0	0	0	0	0.1	0.2	0.3
Erysimum_ischnostylum	0	0	0	2	0	0	0	0	0	0
Euphorbia_boissieriana	0	0	0	0	0.1	0	0	0	0	0
Euphorbia_microsciadia	0	0	0	0	0	0	0.3	0	0	0
Ferula_gummosa	0	0	0	0	0	0	0	0	0	0
Festuca_valesiaca	0	1.7	0	1	0	0	0	0	0.4	5
Fumaria_vaillantii	0	0	0	0	0	0	0	0	0	0
Gagea_gageoides	0	0	0	0	0	0	0	0	0.5	0
Gagea_kunawurensis	0	1	0	0	0	0	0	0	0	0.5
Galium_spurium	0	0.1	0	0	0	0	0	0	0	0
Garhadiolus_hedypnois	0	0	0	0	0	0	0	0	0	0
Geranium_kotschyi	0	0	0	0	0	0	0	0	0	0
Gladiolus_atroviolaceus	0	0	0	0	0	0	0	0	0	0
Henrardia_persica	0	0	0	0	0	0	0	0	0	0
Heteranthelium_piliferum	0	0	0	0	0	0	0	0	0	0
Holosteum_umbellatum	0	0	0	0	0	0	0	0	0	0.1
Hymenocrater_platystegius	0	0	0	0	0	0	0	0	0	0
Hypecoum_pendulum	0	0	0	0	0	0	0	0	0	0
Iris_fosteriana	0	0	0	0	0.2	0	0	0	0	0
Ixiolirion_tataricum	0	0	0	0	0	0	0	0	0	0
Johrenia_platycarpa	0	0	0	0	0	0	0	0	0	1
Jurinea_sintenisii	0	0	0	0	0	0	0	0	0	0
Krascheninnikovia_ceratoides	0	0	0	0	0	0	0	0	0	0
Lactuca_orientalis	0	0	0	0.2	0	1	0	0	0.4	0.1
Lagochilus_cabulicus	0	0	0	0	0	0	0	0	0	0
Lappula_barbata	0	0	0.5	0	0	0	0	0	0	0
Lappula_microcarpa	0	0	0	1	0	0	0.1	0	0	0
Leymus_secalinus	0	0	0	0	0	0	0	0	0	0
Loliolum_subulatum	0	0	0.5	0	0	0	0	0	0	0
Lotus_corniculatus	0	0	0	0	0	0	0	0	0	0
Melica_persica	0	0	5	0	0	0	0	0	0	0
Minuartia_meyeri	0	0	1	0	0	0	0	0.1	0	0
Myosotis_stricta	0	0	0	0	0	0	0.1	0	0	0
Noaea_mucronata	0	0	0	0	0	0	0	0	0.1	0
Onobrychis_cornuta	1	0.3	0	0	0	0	0	0	0	0.5
Onobrychis_verae	0	0	0	0	0	0	0	3	0	0
Perovskia_abrotanoides	0	0	4	0	0	0	0	0	0	0
Phlomoides_binaludensis	0	0	0	0	0	0	0	0	0	0
Poa_bulbosa	0	0	0.5	3	2	0.2	1.5	4	12	0
Polygonum_thymifolium	0	0	0	0	0	1.3	0	0	0	0
Prunus_microcarpa	0	0	1.5	0	0	0	0	0	0	0
Prunus_pseudoprostrata	0	0	0	0	0	0	0	0	0	0
Rhaponticum_repens	0	0	0	0	0	0	0	0	0	0
Rhizocephalus_orientalis	0	0	0	0	0.1	0	0.2	0.1	0.1	0.1
Rochelia_disperma	0	0	0	0	0	0	0	0	0	0
Rochelia_peduncularis	0	0	0	0	0	0	0	0	0	0
Rochelia_persica	0	0	0	0	0	0	0	0	0.1	0
Salvia_chloroleuca	0	0	0	0	0	0	0	0	0	0
Sanguisorba_minor	0	0	0.5	0	0	0	0	0	1.5	0
Scandix_stellata	0	0	0.5	0.1	0	0	0	0	0	0
Scorzonera_leptophylla	0	0.5	0	0	0	0	0	0	0	0
Scutellaria_luteocoerulea	0	0	0	0	0	0	0	0	0	0.1
Senecio_paulsenii	0	0	0	0	0	0	0	0	0	0
Serratula_latifolia	0	0	0	0	0	0	0	0	0	0
Silene_bupleuroides	0	0	0	0	0	0	0	0	0	0
Silene_indeprensa	0	0	0	0	0	0	1	0	0	0
Stachys_lavandulifolia	0	0	0	0	0	0	1.2	0	0	0
Stellaria_alsinoides	0	0	0	0	0	0	0.1	0	0	0
Stipa_hohenackeriana	0	0	0	0	0	0	0	0	0	0
Stipa_holosericea	0	0	0	0	0	0.1	0	0	0	0
Stipa_lessingiana	0	1	0	0	0	0	0.2	0	0.5	0.1
Taeniatherum_caputmedusae	0	0	0	0	0	0	0	0	0	0
Taraxacum_afghanicum	0	0	0	0	0	0	0	0	1	0
Thalictrum_isopyroides	0	0	0	0	0	0	0	0	0	0
Tulipa_micheliana	0	0	0	0	0	0	0	0	0	0
Verbascum_cheiranthifolium	0.1	2.7	4	0.5	0	1	0.1	0.3	2.3	0.5
Veronica_arguteserrata	1	0	0	0	0	0	0	0	0	0
Veronica_campylopoda	0	0	0.5	0	0	0	0.7	0	0	0
Veronica_rubrifolia	0	0.5	0	0	0	0	0	0.1	0.1	0
Vicia_subvillosa	0	0	0	0	3	0	0	0	0	0
Viola_occulta	0	0	0	0	0	0	0	0.1	0	0

Phlomis cancellata
Kelilagh

CFS

Acanthophyllum_sp_	0	1	0	0	0	1	0	0	2	0	0	0	0	0	0
Achillea_biebersteinii	0	0	0	0	0	0	0	0	0	0	0	0	0	0	0
Achillea_wilhelmsii	1	0	0	0	0	0	1	0	0	0	0	0	0	0	0
Clinopodium_graveolens	0	0	0	0	0	0	0	0	0	0	1	0	0	0	0
Agropyron_intermedium	0	0	0	0	0	0	0	1	1	0	0	0	0	1	0
Alhagi_maurorum	0	0	0	0	0	0	0	0	0	0	0	0	0	0	0
Allium_kuhsorkhense	0	0	0	0	1	0	0	0	0	0	0	0	0	0	0
Alyssum_dasycarpum	0	0	0	0	0	0	1	0	0	0	0	0	0	0	0
Alyssum_linifolium	0	0	0	0	0	0	0	0	0	0	0	0	0	0	0
Alyssum_stapfii	0	0	0	0	0	0	0	0	0	0	0	0	0	0	0
Alyssum_sp_	0	0	0	0	0	0	0	0	0	0	0	0	0	0	0
Androsace_maxima	0	0	0	0	0	0	0	0	0	0	1	0	1	0	0
Artemisia_scoparia	0	0	0	0	0	0	0	0	0	0	0	0	1	0	0
Artemisia_tournefortiana	0	0	0	0	0	0	0	0	0	0	0	0	0	0	0
Astragalus_brevidens	0	0	0	0	0	0	0	0	0	0	0	0	0	0	0
Astragalus_campylorhynchus	0	0	0	0	0	0	0	0	0	0	0	0	0	0	0
Astragalus_chrysostachys	0	0	0	0	0	0	0	0	0	0	0	0	0	0	0
Astragalus_commixtus	0	0	0	0	0	0	0	0	0	0	0	0	0	0	0
Astragalus_verus	0	0	0	0	0	0	0	0	0	1	0	0	0	0	0
Bromus_danthoniae	1	0	0	0	1	0	0	0	0	0	0	1	0	0	0
Callipeltis_cucullaris	0	0	0	0	0	0	0	0	0	0	1	0	0	0	0
Carex_stenophylla	0	0	0	0	0	1	0	0	0	0	0	0	0	0	0
Centaurea_virgata	0	0	0	0	0	0	0	0	0	0	0	0	0	0	0
Ceratocephala_falcata	0	0	0	0	0	0	0	0	0	0	0	0	0	0	0
Ceratocephala_testiculata	0	0	0	0	0	0	0	0	0	0	0	0	0	0	0
Chenopodium_glaucum	0	0	0	0	0	0	0	0	0	0	0	0	0	0	0
Cirsium_arvense	0	0	0	0	0	0	0	0	0	0	0	0	0	0	0
Colchicum_kotschyi	0	0	0	0	0	0	0	0	0	0	0	0	0	1	0
Convolvulus_arvensis	0	0	0	0	0	0	0	0	0	0	0	0	0	0	0
Convolvulus_lineatus	0	0	0	0	0	0	0	0	0	0	0	0	1	0	0
Cotoneaster_kotschyi	0	0	0	0	0	0	0	0	0	0	0	0	0	0	0
Cousinia_sp_	1	1	0	0	1	0	0	0	0	0	0	0	0	0	0
Crucianella_gilanica	0	0	0	0	0	0	0	0	0	0	0	0	0	0	0
Cymbolaena_griffithii	0	1	0	1	0	0	0	0	0	0	1	0	0	0	0
Dianthus_polylepis	0	0	0	0	0	0	0	0	0	0	0	0	0	0	0
Elwendia_chaerophylloides	0	0	0	0	0	0	0	0	0	0	0	0	0	0	1
Eremurus_olgae	1	0	1	0	0	0	1	0	0	0	0	0	0	0	0
Eremurus_spectabilis	0	0	0	0	0	0	0	0	0	1	0	0	0	0	0
Eryngium_bungei	0	0	0	1	0	1	0	0	0	0	0	1	0	0	0
Euphorbia_buhsei	0	0	0	0	0	0	0	0	0	0	0	0	0	0	0
Galium_humifusum	1	0	0	0	0	0	0	1	0	0	0	0	0	0	1
Galium_sp_	0	0	0	0	0	1	0	0	0	0	0	0	0	0	0
Gundelia_tournefortii	0	0	1	1	1	0	0	0	0	0	0	0	0	0	0
Hymenocrater_elegans	0	0	0	0	0	0	0	0	0	0	1	0	0	1	0
Hyoscyamus_reticulatus	0	0	0	0	0	0	0	0	0	0	0	0	0	0	0
Ixiolirion_tataricum	0	0	0	0	0	0	0	0	0	0	1	0	0	0	0
Lactuca_orientalis	1	0	0	1	0	0	0	0	0	0	0	0	0	0	0
Lappula_barbata	0	0	0	0	0	0	0	0	0	0	0	0	0	0	0
Lappula_sinaica	0	0	0	0	0	0	0	0	0	0	0	0	0	0	0
Marrubium_anisodon	0	0	0	0	0	0	0	0	0	0	0	0	0	0	0
Mesostemma_kotschyana	0	0	0	0	0	0	0	1	0	0	0	0	0	0	0
Nepeta_bracteata	0	0	0	0	0	0	0	0	0	0	0	0	0	0	0
Nepeta_glomerulosa	0	0	1	0	0	0	0	0	0	0	0	0	0	0	0
Onosma_longiloba	0	0	0	0	0	0	0	0	0	0	0	0	0	0	0
Poa_bulbosa	1	0	0	0	0	1	0	0	0	1	0	0	1	0	1
Polygonum_afghanicum	0	0	0	0	0	0	0	0	0	0	0	0	0	0	0
Polygonum_polycnemoides	1	0	0	0	0	0	0	0	0	0	0	0	0	0	0
Rochelia_bungei	0	0	0	0	0	0	0	0	0	0	0	0	0	0	0
Rosa_beggeriana	0	0	0	0	0	0	0	1	0	0	0	0	0	0	1
Rosa_persica	0	0	0	0	0	0	0	0	0	0	0	0	0	0	0
Scandix_stellata	0	0	0	0	0	0	0	0	0	0	0	0	0	1	0
Scrophularia_scoparia	0	0	0	1	0	0	0	0	0	0	0	0	0	0	0
Scrophularia_striata	0	0	0	0	0	0	0	0	0	0	0	0	0	0	0
Sisymbrium_loeselii	0	0	0	0	0	0	0	0	0	0	0	0	0	0	0
Stipa_hohenackeriana	0	0	0	0	0	0	0	0	0	0	0	0	0	0	0
Taraxacum_sp_	0	0	0	0	0	0	0	0	0	0	0	0	0	0	0
Thalictrum_isopyroides	0	0	0	0	0	0	0	0	0	0	0	1	0	0	0
Tragopogon_collinus	0	0	0	0	0	0	0	0	0	0	0	0	0	0	0
Veronica_sp_	0	0	0	0	0	0	1	0	0	0	0	0	0	0	1
Verbascum_songaricum	0	0	0	0	0	0	0	1	0	0	0	0	0	0	0
Verbascum_sp_	0	0	0	0	0	0	0	0	0	0	0	0	0	0	0
Ziziphora_tenuior	0	0	0	0	0	0	0	0	0	0	0	0	0	0	0

WFS

Acanthophyllum_sp_	1	0	1	0	0	0	0	0	0	0	0	1	0	0
Achillea_biebersteinii	0	0	0	0	0	0	1	0	0	0	0	0	0	0
Achillea_wilhelmsii	0	0	0	0	0	0	0	0	1	0	0	0	0	0
Clinopodium_graveolens	0	0	0	0	0	0	0	0	0	0	0	0	0	0
Agropyron_intermedium	0	0	0	0	0	0	0	1	0	0	0	0	0	0
Alhagi_maurorum	0	0	0	0	0	0	0	0	0	1	0	0	0	0
Allium_kuhsorkhense	0	0	0	0	0	0	0	0	0	0	0	0	0	0
Alyssum_dasycarpum	0	0	0	0	0	0	0	0	0	0	0	0	0	0
Alyssum_linifolium	1	0	0	0	0	1	1	0	0	0	0	0	0	0
Alyssum_stapfii	0	0	0	0	0	0	0	0	0	0	1	0	0	0
Alyssum_sp_	0	0	0	0	0	0	0	0	0	0	0	0	1	0
Androsace_maxima	0	0	0	0	0	0	0	0	0	0	0	0	1	0
Artemisia_scoparia	0	0	0	0	0	0	0	0	0	0	1	1	0	0
Artemisia_tournefortiana	0	0	0	0	0	0	0	0	0	0	0	0	1	0
Astragalus_brevidens	0	0	0	0	0	0	0	0	1	0	0	0	0	1
Astragalus_campylorhynchus	0	0	0	0	0	0	0	0	0	1	0	0	0	0
Astragalus_chrysostachys	0	0	0	0	0	0	0	0	0	0	1	0	0	0
Astragalus_commixtus	0	1	0	0	0	0	0	0	0	0	0	0	0	0
Astragalus_verus	0	0	0	0	0	0	0	0	0	0	0	0	0	0
Bromus_danthoniae	1	1	1	1	1	0	0	0	0	1	1	1	0	0
Callipeltis_cucullaris	0	0	0	0	0	0	0	0	0	0	0	0	0	0
Carex_stenophylla	0	1	0	0	0	0	0	0	0	0	0	0	0	0
Centaurea_virgata	0	0	0	0	1	0	0	0	0	0	0	0	0	0
Ceratocephala_falcata	0	1	0	0	0	1	1	0	0	0	0	0	0	0
Ceratocephala_testiculata	0	0	0	0	0	1	0	0	0	0	0	0	0	0
Chenopodium_glaucum	1	0	0	0	0	0	0	0	0	0	0	0	0	0
Cirsium_arvense	0	0	0	0	0	0	0	0	0	1	0	0	0	0
Colchicum_kotschyi	0	0	0	0	0	0	0	0	0	0	0	0	0	0
Convolvulus_arvensis	0	0	0	0	0	0	0	0	0	0	1	0	0	0
Convolvulus_lineatus	0	0	0	0	0	0	0	0	1	0	0	1	0	0
Cotoneaster_kotschyi	0	0	0	0	0	0	0	0	0	0	0	0	0	1
Cousinia_sp_	0	0	0	1	0	0	0	0	0	0	0	0	0	0
Crucianella_gilanica	0	0	0	0	0	0	0	0	0	1	0	0	0	0
Cymbolaena_griffithii	0	0	0	0	0	0	0	0	0	0	0	0	0	0
Dianthus_polylepis	0	0	0	0	1	0	0	0	0	0	0	0	0	0
Elwendia_chaerophylloides	0	0	0	0	0	0	0	0	0	0	0	0	0	0
Eremurus_olgae	0	0	0	0	0	0	0	0	0	0	0	0	0	0
Eremurus_spectabilis	0	0	0	0	0	0	0	0	0	0	0	0	0	0
Eryngium_bungei	0	0	0	0	0	0	0	0	0	0	0	0	0	0
Euphorbia_buhsei	1	0	0	0	0	0	0	0	0	0	0	0	0	0
Galium_humifusum	0	0	0	0	0	1	0	0	0	0	0	0	0	0
Galium_sp_	0	0	0	0	0	0	0	0	0	1	0	0	0	0
Gundelia_tournefortii	0	1	1	1	1	0	0	0	0	0	0	0	0	0
Hymenocrater_elegans	0	0	1	0	0	0	0	0	0	0	0	0	0	0
Hyoscyamus_reticulatus	0	0	1	0	0	0	0	0	0	0	0	0	0	0
Ixiolirion_tataricum	0	0	0	0	0	0	0	0	0	0	0	0	0	0
Lactuca_orientalis	0	1	0	1	1	1	1	0	1	0	0	0	1	0
Lappula_barbata	0	0	0	0	0	0	0	0	0	1	0	0	0	0
Lappula_sinaica	0	0	0	0	0	0	0	1	0	0	0	0	0	0
Marrubium_anisodon	0	0	0	0	0	1	0	0	0	0	0	0	0	0
Mesostemma_kotschyana	0	0	0	0	0	0	0	1	0	0	0	0	0	0
Nepeta_bracteata	1	0	0	0	0	0	0	0	0	0	0	0	0	0
Nepeta_glomerulosa	0	0	0	0	0	0	0	0	0	0	0	0	0	0
Onosma_longiloba	0	0	0	0	0	0	1	0	0	0	0	0	0	0
Poa_bulbosa	0	1	1	0	0	1	0	1	1	0	1	1	1	0
Polygonum_afghanicum	0	0	0	0	1	0	0	0	0	0	0	0	0	0
Polygonum_polycnemoides	0	0	0	1	0	0	0	0	0	0	0	0	0	0
Rochelia_bungei	0	0	0	0	0	0	0	0	0	1	0	0	0	0
Rosa_beggeriana	0	0	0	0	0	0	0	0	0	0	0	0	0	0
Rosa_persica	0	0	0	0	0	0	0	1	1	0	0	0	0	0
Scandix_stellata	0	0	0	0	0	0	0	0	0	0	0	0	0	0
Scrophularia_scoparia	0	0	0	0	0	0	0	0	0	0	0	0	0	0
Scrophularia_striata	0	0	0	0	0	0	0	0	0	1	0	0	0	0
Sisymbrium_loeselii	0	0	0	0	0	0	0	0	0	1	0	0	0	0
Stipa_hohenackeriana	0	0	0	0	0	0	0	0	0	0	0	0	0	1
Taraxacum_sp_	0	0	0	0	0	0	0	0	1	0	0	0	0	0
Thalictrum_isopyroides	0	0	0	0	0	0	0	0	0	0	0	0	0	0
Tragopogon_collinus	0	1	0	0	0	0	0	0	0	0	0	0	0	0
Veronica_sp_	1	0	0	0	0	0	0	0	0	0	0	0	0	0
Verbascum_songaricum	0	0	0	0	0	0	0	0	1	0	0	0	0	1
Verbascum_sp_	0	0	0	0	0	0	1	0	0	0	0	0	0	0
Ziziphora_tenuior	0	0	0	1	0	0	0	0	0	0	0	0	0	0

Phlomis cancellata
Najafi

CFS

Acantholimon_pterostegium	0	0	1	0	0	0	0	0
Acanthophyllum_sordidum	1	0	0	0	1	0	0	0
Aethionema_carneum	0	0	0	0	0	0	0	0
Allium_xiphopetalum	0	0	0	0	0	0	2	0
Alyssum_desertorum	0	0	8	0	0	0	0	0
Alyssum_stapfii	0	0	0	0	0	0	0	2
Anagallis_arvensis	0	0	0	0	0	0	0	0
Artemisia_scoparia	0	2	0	0	0	0	0	0
Astragalus_oxyglottis	0	0	0	0	0	0	0	0
Bellevalia_saviczii	0	0	0	0	0	0	0	0
Boissiera_squarrosa	0	0	0	0	0	0	0	3
Bromus_danthoniae	0	0	0	0	0	0	0	0
Bromus_tectorum	0	0	0	0	5	0	2	0
Callipeltis_cucullaria	4	0	0	0	0	0	0	0
Campanula_khorasanica	0	0	0	0	0	0	0	0
Caroxylon_incanescens	0	0	0	0	0	0	0	0
Centaurea_virgata	0	0	0	1	0	0	0	0
Cerastium_dichotomum	0	0	0	0	0	8	0	0
Clinopodium_graveolens	6	0	0	0	2	0	0	0
Conringia_orientalis	0	0	0	2	0	0	0	0
Crepis_kotschyana	3	0	0	0	0	0	0	0
Cynodon_dactylon	0	0	0	0	0	0	0	0
Elwendia_persicum	0	0	0	0	0	1	0	0
Enneapogon_persicus	0	0	0	0	0	0	3	4
Eremurus_spectabilis	0	0	0	0	0	0	0	0
Euphorbia_inderiensis	0	0	0	0	0	0	0	0
Euphorbia_spinidens	0	0	0	0	0	0	1	0
Euphorbia_szovitsii	0	0	0	0	0	0	0	0
Filago_arvensis	0	0	0	0	0	0	0	0
Fritillaria_gibbosa	0	0	0	0	0	0	0	0
Gagea_reticulata	0	0	0	0	0	0	0	0
Gypsophila_pilosa	0	0	0	0	0	0	0	0
Heterocaryum_subsessile	0	0	0	0	0	0	0	0
Iris_kopetdagensis	0	0	0	1	0	0	0	0
Ixiolirion_tataricum	0	0	0	2	0	0	0	0
Lactuca_glauciifolia	0	0	0	5	0	0	0	0
Lactuca_orientalis	0	0	0	0	0	0	0	0
Lactuca_persica	0	0	0	0	0	0	0	0
Lactuca_undulata	3	0	0	0	0	0	0	0
Lappula_barbata	0	0	0	0	0	0	1	0
Mesostemma_kotschyanum	0	0	0	0	2	0	0	0
Melica_persica	0	0	0	0	0	0	0	0
Nigella_integrifolia_	0	0	0	0	0	4	0	0
Perovskia_abrotanoides	0	0	0	0	0	0	0	0
Poa_bulbosa	0	5	10	0	3	6	3	4
Polygonum_paronychioides	0	1	0	0	0	0	0	0
Polygonum_polycnemoides	0	0	0	0	0	0	0	0
Rumex_tuberosus	0	0	0	0	0	0	0	0
Scabiosa_olivieri	0	0	0	1	0	0	0	0
Scandix_stellata	0	0	2	0	0	6	0	0
Scrophularia_variegata	0	0	1	0	0	0	0	0
Silene_latifolia	0	0	0	0	1	0	0	0
Stipa_hohenackeriana	0	0	0	0	0	0	0	1
Taeniatherum_caputmedusae	0	0	0	0	0	0	0	0
Thalictrum_sultanabadense	0	0	0	0	0	0	0	0
Tragopogon_collinus	0	0	0	0	0	0	0	0
Tripleurospermum_disciforme	0	0	0	0	0	0	0	0
Trigonella_monantha	0	0	0	0	0	5	0	0
Veronica_anagallisaquatica	0	0	0	0	0	0	0	0
Veronica_ferganica	0	0	0	0	0	0	0	0
Verbena_officinalis	0	0	0	0	0	0	0	0
Verbascum_songaricum	0	1	0	0	0	0	0	0
Vicia_sativa	0	0	0	0	0	0	0	0
Ziziphora_clinopodioides	0	0	0	1	0	0	0	0
Ziziphora_tenuior	0	0	0	0	0	0	0	0

WFS

Acantholimon_pterostegium	0	0	0	0	0	0	0	0
Acanthophyllum_sordidum	0	0	0	0	0	0	0	0
Aethionema_carneum	0	5	0	2	0	0	0	0
Allium_xiphopetalum	0	0	0	0	0	0	0	0
Alyssum_desertorum	0	0	8	4	6	0	4	0
Alyssum_stapfii	0	0	0	0	0	0	0	9
Anagallis_arvensis	0	2	0	0	0	0	0	0
Artemisia_scoparia	0	0	0	0	0	0	0	0
Astragalus_oxyglottis	0	0	0	0	2	0	0	0
Bellevalia_saviczii	0	0	0	1	0	0	0	0
Boissiera_squarrosa	0	0	0	0	0	0	0	0
Bromus_danthoniae	0	0	0	0	0	0	0	4
Bromus_tectorum	0	0	0	0	0	0	0	0
Callipeltis_cucullaria	0	0	0	0	0	0	0	0
Campanula_khorasanica	0	6	0	0	0	0	0	0
Caroxylon_incanescens	0	0	0	0	0	0	1	0
Centaurea_virgata	0	0	0	0	0	0	0	0
Cerastium_dichotomum	0	0	0	0	0	0	0	0
Clinopodium_graveolens	0	0	0	0	0	0	0	0
Conringia_orientalis	0	0	0	0	0	0	0	0
Crepis_kotschyana	0	0	0	0	0	0	5	0
Cynodon_dactylon	1	0	0	0	0	0	0	0
Elwendia_persicum	0	0	0	0	0	0	0	0
Enneapogon_persicus	0	0	0	0	0	0	0	0
Eremurus_spectabilis	0	0	0	0	1	0	0	0
Euphorbia_inderiensis	0	0	0	0	0	0	1	0
Euphorbia_spinidens	0	0	0	0	0	3	0	0
Euphorbia_szovitsii	0	0	0	0	3	0	0	0
Filago_arvensis	0	0	0	0	0	4	0	0
Fritillaria_gibbosa	0	0	0	2	0	0	0	0
Gagea_reticulata	0	0	0	3	0	0	0	0
Gypsophila_pilosa	2	0	0	0	0	0	0	0
Heterocaryum_subsessile	0	0	6	0	0	0	0	0
Iris_kopetdagensis	0	0	0	0	0	0	0	0
Ixiolirion_tataricum	0	0	0	0	0	0	0	0
Lactuca_glauciifolia	0	0	0	0	0	0	0	0
Lactuca_orientalis	0	0	0	1	0	0	0	0
Lactuca_persica	1	0	0	0	0	0	0	0
Lactuca_undulata	0	0	0	0	0	0	0	0
Lappula_barbata	0	0	0	0	0	0	0	0
Mesostemma_kotschyanum	0	0	0	0	0	0	0	0
Melica_persica	0	0	0	0	0	0	0	6
Nigella_integrifolia_	0	0	0	0	0	0	0	0
Perovskia_abrotanoides	0	0	0	0	0	0	0	3
Poa_bulbosa	0	0	3	3	5	0	6	0
Polygonum_paronychioides	0	0	0	0	0	2	0	0
Polygonum_polycnemoides	0	0	0	2	0	0	0	0
Rumex_tuberosus	0	0	0	0	1	0	0	0
Scabiosa_olivieri	0	0	0	0	0	0	0	0
Scandix_stellata	0	0	10	0	0	0	0	0
Scrophularia_variegata	0	0	0	0	0	0	0	0
Silene_latifolia	0	0	0	0	0	0	0	0
Stipa_hohenackeriana	0	0	0	0	0	0	0	0
Taeniatherum_caputmedusae	0	0	0	0	2	0	0	0
Thalictrum_sultanabadense	0	0	0	0	0	0	0	10
Tragopogon_collinus	0	1	0	0	0	0	0	0
Tripleurospermum_disciforme	0	0	0	0	0	0	0	1
Trigonella_monantha	0	0	6	0	0	0	0	0
Veronica_anagallisaquatica	0	4	0	0	0	0	0	0
Veronica_ferganica	2	0	0	0	0	0	0	0
Verbena_officinalis	3	0	0	0	0	0	0	0
Verbascum_songaricum	0	0	0	0	0	0	0	0
Vicia_sativa	0	0	3	0	0	0	0	0
Ziziphora_clinopodioides	0	0	0	0	0	0	3	0
Ziziphora_tenuior	4	0	0	0	0	0	0	0

Phlomis cancellata
Najafi

CFS

Acanthophyllum_glandulosum	0	0	0	0	1	0	0	0	0	0	0	0	0	0	0	0
Acanthophyllum_sordidum	0	0	0	0	0	0	0	2	0	0	0	0	0	0	0	0
Acanthophyllum_sp_	0	0	0	0	0	0	0	0	0	0	0	0	2	0	0	0
Achillea_wilhelmsii	0	0	0	0	0	0	0	0	0	0	0	0	0	0	0	0
Clinopodium_graveolens	0	0	0	0	0	0	0	0	0	0	0	0	0	1	0	0
Agropyron_intermedium	5	0	0	0	0	1	0	0	0	0	0	0	0	0	1	0
Alyssum_dasycarpum	0	0	0	0	0	0	0	0	0	0	2	0	0	0	0	0
Alyssum_harputicum	0	0	0	0	0	0	0	0	0	0	0	0	0	2	0	0
Alyssum_linifolium	0	0	0	0	0	0	0	0	0	0	0	0	0	0	0	0
Alyssum_minus	0	0	0	1	0	0	0	0	0	0	0	0	0	0	0	0
Alyssum_niveum	0	0	0	0	0	0	0	0	0	0	0	0	0	0	1	0
Amygdalus_spinosissima	0	0	0	0	0	0	0	0	1	0	0	2	0	0	0	0
Androsace_maxima	0	0	0	0	0	1	0	3	0	0	0	0	0	0	0	0
Arrhenatherum_kotschyi	0	0	0	0	0	0	0	0	0	0	0	1	0	0	0	0
Artemisia_tournefortiana	0	0	0	0	0	0	0	0	0	0	0	0	0	0	0	1
Asperula_glomerata	0	0	0	0	0	1	0	0	0	0	0	0	0	0	0	0
Astragalus_brevidens	0	0	0	0	0	0	1	0	0	0	0	0	0	0	0	0
Astragalus_campylotrichus	0	0	0	0	0	0	0	0	0	0	0	0	0	0	0	0
Astragalus_gompholobium	0	0	0	0	0	0	0	0	0	0	0	0	0	0	1	0
Astragalus_lycioides	0	0	0	0	0	0	0	0	0	0	0	0	0	0	0	0
Astragalus_reticulatus	0	0	0	0	0	2	0	0	0	0	0	0	0	0	0	0
Astragalus_sieversianus	0	0	0	0	0	0	0	0	0	0	0	0	0	0	0	0
Astragalus_verus	0	0	0	0	0	0	0	0	1	0	0	0	1	0	0	0
Avena_barbata	0	0	0	0	0	0	0	0	0	0	0	0	0	0	0	0
Biebersteinia_multifida	0	0	0	0	0	0	0	0	0	0	0	0	0	0	1	0
Bromus_danthoniae	0	0	4	6	0	0	0	0	0	0	0	0	0	0	0	0
Callipeltis_cucullaris	0	0	0	0	0	0	0	0	0	0	0	0	0	0	0	0
Carex_sp_	0	0	0	0	0	0	0	0	5	0	0	0	0	0	0	0
Carex_stenophylla	0	0	0	0	0	0	0	0	0	0	0	0	0	0	0	0
Centaurea_virgata	0	0	0	0	0	0	0	0	0	0	1	0	0	0	0	0
Cirsium_sorocephalum	0	2	0	0	0	0	0	0	0	0	0	0	0	0	0	0
Cleome_coluteoides	0	0	0	0	0	0	0	0	0	0	0	0	0	0	0	0
Colchicum_kotschyi	0	0	0	0	0	0	0	0	0	0	0	0	0	0	0	0
Convolvulus_arvensis	0	0	0	10	0	0	0	0	0	0	0	0	0	0	0	0
Cousinia_sp_	0	0	1	0	0	0	0	0	0	0	0	0	0	0	0	0
Cousinia_microcarpa	0	0	0	0	0	0	0	0	0	0	0	0	0	0	0	0
Descurainia_sophia	0	0	0	0	0	0	0	0	0	0	0	0	0	0	0	0
Dianthus_polylepis	0	0	0	0	0	0	0	0	0	0	0	0	0	0	0	0
Elymus_hispidus	0	0	0	0	0	0	0	0	0	0	0	0	0	0	0	0
Eremopyrum_bonaepartis	0	0	0	0	0	0	0	0	0	0	0	0	2	0	0	0
Eremurus_olgae	0	0	0	0	0	0	0	0	0	0	0	0	0	2	0	0
Eremurus_spectabilis	0	0	0	0	0	0	0	2	0	0	0	0	0	0	0	0
Eremurus_stenophyllus	0	0	0	0	0	0	1	0	0	0	0	0	0	0	0	0
Eryngium_billardierei	0	0	2	0	0	0	0	0	0	0	0	0	0	0	0	0
Eryngium_bungei	0	0	0	0	0	0	0	0	0	0	0	0	0	0	0	0
Ferula_ovina	0	0	0	0	0	0	0	0	0	1	1	0	0	0	0	0
Galium_humifusum	0	0	0	0	0	0	0	0	0	0	0	0	6	0	0	0
Galium_sp_	0	0	0	0	0	0	0	0	0	0	0	0	0	0	0	0
Gentiana_olivieri	0	0	0	0	0	4	0	0	0	0	0	0	0	0	0	0
Gundelia_tournefortii	0	0	0	2	0	1	1	0	0	0	0	1	0	0	0	0
Hordeum_murinum	0	0	0	0	0	0	0	0	0	0	0	0	0	0	0	0
Hymenocrater_elegans	0	0	0	0	0	0	0	0	0	0	0	0	0	1	0	1
Hypericum_scabrum	0	0	0	0	0	0	0	0	0	0	0	0	0	1	0	1
Iris_songarica	0	2	0	0	0	0	0	0	0	0	0	0	0	0	0	0
Ixiolirion_tataricum	0	0	0	0	0	0	0	0	0	1	0	0	0	0	0	0
Lactuca_orientalis	0	0	0	0	0	0	0	0	0	0	0	0	0	0	0	0
Lallemantia_royleana	0	0	0	0	0	1	0	0	0	0	0	0	0	0	0	0
Lappula_sinaica	0	0	0	0	0	0	0	0	0	0	0	0	0	0	0	0
Lepidium_draba	0	0	0	0	0	0	0	0	0	0	0	0	0	0	0	0
Lepyrodiclis_sp_	0	0	0	0	0	0	0	0	0	0	0	0	0	0	0	0
Marrubium_anisodon	0	0	0	0	0	0	0	0	0	1	0	0	1	0	2	0
Medicago_lupulina	0	0	0	0	0	0	0	0	0	0	0	0	0	0	0	0
Medicago_sativa	0	0	0	0	0	0	1	0	0	0	0	0	0	0	0	0
Nepeta_saccharata	0	0	0	0	0	0	0	0	0	0	0	0	0	0	0	0
Onobrychis_cornuta	0	0	0	0	0	0	0	0	0	0	0	0	0	0	0	0
Papaver_dubium	0	0	0	0	0	0	0	0	0	0	0	0	0	0	0	0
Parietaria_judaica	0	0	0	0	0	0	0	0	0	0	0	0	0	0	0	0
Poa_bulbosa	0	0	0	5	20	0	0	10	6	0	0	0	0	4	4	0
Polygonum_polycnemoides	0	0	3	0	0	0	0	0	0	0	0	0	0	0	0	0
Rosa_pumila	0	0	0	0	0	0	0	0	0	0	1	0	0	0	0	0
Sanguisorba_minor	0	0	0	0	0	0	0	0	0	1	0	1	1	0	0	0
Scandix_stellata	0	0	0	0	0	0	0	0	0	0	0	0	0	0	0	0
Scorzonera_raddeana	0	0	0	0	0	0	0	0	0	0	1	0	0	0	0	0
Scrophularia_scoparia	0	0	0	0	0	0	0	0	0	0	0	0	0	2	0	0
Silene_swertiaefolia	0	0	0	0	0	0	0	0	0	0	0	0	0	0	2	0
Stellaria_alsinoides	0	0	0	0	0	0	0	0	0	0	2	0	0	0	0	0
Stipa_hohenackeriana	0	2	0	0	0	0	0	0	0	0	0	0	0	0	0	0
Taeniatherum_caputmedusae	0	8	0	0	0	0	20	0	0	0	0	0	0	0	0	0
Tanacetum_khorassanicum	0	0	0	0	0	0	0	0	0	0	0	0	0	0	0	2
Tragopogon_collinus	0	0	0	0	0	0	0	0	0	1	0	0	0	0	0	0
Veronica_sp_	0	0	0	8	0	0	0	0	0	0	0	0	0	0	3	0
Verbascum_songaricum	0	0	0	0	2	0	0	0	0	0	0	0	0	0	0	0
Verbascum_sp_	0	0	0	0	0	0	0	0	1	0	0	0	0	0	0	0
Ziziphora_clinopodioides	0	0	0	0	0	0	0	0	0	0	0	0	0	0	0	0
Ziziphora_tenuior	0	0	0	0	0	0	0	0	0	0	0	3	0	0	0	4

WFS

Acanthophyllum_glandulosum	0	0	0	0	0	0	0	0	0	0	0	0	0	0	0	0	0	0
Acanthophyllum_sordidum	0	0	0	0	0	0	0	0	0	0	0	0	0	0	3	0	0	0
Acanthophyllum_sp_	0	0	0	0	0	0	0	0	2	2	0	0	0	1	0	2	0	0
Achillea_wilhelmsii	0	0	0	0	0	0	0	0	4	0	0	0	0	0	0	0	0	0
Clinopodium_graveolens	0	0	0	0	0	0	0	0	0	0	0	0	0	0	0	0	0	0
Agropyron_intermedium	0	0	0	0	0	0	0	5	0	0	0	0	0	0	0	3	0	0
Alyssum_dasycarpum	0	0	3	0	0	0	0	0	0	0	0	0	3	0	0	0	10	0
Alyssum_harputicum	0	0	0	0	0	0	0	0	0	0	0	0	0	0	0	0	0	0
Alyssum_linifolium	0	4	0	0	0	0	8	0	0	0	0	0	0	0	0	0	0	0
Alyssum_minus	0	0	0	0	0	0	0	0	0	0	0	0	0	0	0	0	0	0
Alyssum_niveum	0	0	0	0	0	0	0	0	0	0	0	0	0	0	0	0	0	0
Amygdalus_spinosissima	0	0	0	0	0	0	0	0	0	0	0	0	0	0	0	0	0	0
Androsace_maxima	0	0	0	0	0	0	0	0	1	0	0	0	0	0	0	0	0	0
Arrhenatherum_kotschyi	0	0	0	0	0	0	0	0	0	0	0	0	0	0	0	0	0	0
Artemisia_tournefortiana	0	0	0	0	0	0	0	0	0	0	0	0	0	0	0	0	0	1
Asperula_glomerata	0	0	0	0	0	0	0	0	0	0	0	0	0	0	0	0	0	0
Astragalus_brevidens	0	0	0	0	0	0	0	0	0	0	0	0	0	0	0	0	0	0
Astragalus_campylotrichus	0	0	0	0	0	0	0	0	0	0	2	0	0	0	0	0	0	0
Astragalus_gompholobium	0	0	0	0	0	0	0	0	0	0	1	0	0	0	0	2	0	0
Astragalus_lycioides	0	2	0	0	0	0	0	0	0	0	0	0	0	0	0	0	0	0
Astragalus_reticulatus	0	0	0	0	0	0	0	0	1	0	0	0	0	0	0	0	0	0
Astragalus_sieversianus	0	0	1	0	0	0	0	0	0	0	0	0	0	0	0	0	0	0
Astragalus_verus	0	0	0	0	0	0	0	0	0	0	0	0	0	0	0	0	3	0
Avena_barbata	0	0	0	0	0	0	0	0	0	0	0	0	0	3	0	0	0	0
Biebersteinia_multifida	0	0	0	0	0	0	0	0	0	0	0	2	0	0	0	0	0	0
Bromus_danthoniae	0	0	0	15	0	8	8	8	0	0	0	0	0	0	0	0	0	0
Callipeltis_cucullaris	0	0	0	0	0	0	0	0	0	0	3	0	0	0	0	0	0	0
Carex_sp_	0	7	0	0	0	0	0	0	0	0	0	0	0	0	0	0	0	0
Carex_stenophylla	7	0	0	0	0	0	0	0	0	0	0	0	0	0	0	0	0	0
Centaurea_virgata	0	0	0	0	2	0	0	0	0	0	0	1	0	0	0	0	0	0
Cirsium_sorocephalum	0	0	0	0	0	0	0	0	0	0	0	0	0	0	0	0	0	0
Cleome_coluteoides	0	0	0	0	0	0	0	0	0	0	0	1	0	0	0	0	0	0
Colchicum_kotschyi	0	0	0	0	0	0	0	0	0	0	0	0	0	1	0	0	0	0
Convolvulus_arvensis	0	0	0	0	0	0	0	0	0	0	0	0	0	0	0	0	0	0
Cousinia_sp_	0	0	0	0	0	0	1	1	0	0	0	0	0	0	0	0	0	0
Cousinia_microcarpa	0	0	0	2	0	0	0	0	0	0	0	0	0	0	0	0	0	0
Descurainia_sophia	0	0	0	0	0	10	0	0	0	0	0	0	0	0	0	0	0	0
Dianthus_polylepis	0	0	0	0	0	0	0	1	0	0	0	0	0	0	0	0	0	0
Elymus_hispidus	0	0	0	0	0	0	0	0	0	0	0	0	2	0	0	0	0	0
Eremopyrum_bonaepartis	0	0	0	0	0	0	0	0	0	0	0	0	0	0	0	0	0	0
Eremurus_olgae	0	0	0	0	0	1	0	1	0	0	0	0	0	0	0	0	0	0
Eremurus_spectabilis	0	0	0	0	0	0	1	0	0	0	0	0	0	1	0	0	0	0
Eremurus_stenophyllus	0	0	0	0	0	0	0	0	0	0	0	0	0	0	0	0	0	0
Eryngium_billardierei	0	0	0	1	0	0	0	0	0	0	0	0	0	0	0	0	0	0
Eryngium_bungei	0	0	0	0	2	0	0	0	0	1	0	0	0	0	0	0	0	0
Ferula_ovina	0	0	0	0	0	0	0	0	0	0	0	0	0	0	0	0	0	0
Galium_humifusum	0	0	0	0	0	0	0	0	0	0	0	0	0	0	0	0	0	0
Galium_sp_	0	0	0	0	0	0	0	0	0	0	0	0	0	0	0	0	3	0
Gentiana_olivieri	0	0	0	0	3	0	0	0	0	0	0	0	0	0	0	0	0	0
Gundelia_tournefortii	0	0	1	0	0	0	0	0	0	1	2	0	0	0	0	0	0	0
Hordeum_murinum	0	0	0	5	0	10	0	0	0	0	0	0	0	0	0	0	0	0
Hymenocrater_elegans	0	0	0	0	0	0	0	0	0	0	0	0	0	0	2	0	0	0
Hypericum_scabrum	0	0	0	0	0	0	0	0	0	0	0	0	0	0	0	0	0	0
Iris_songarica	0	0	0	0	0	0	0	0	0	0	0	0	0	0	0	0	0	0
Ixiolirion_tataricum	0	0	0	0	0	0	0	0	0	0	0	0	0	0	0	0	0	0
Lactuca_orientalis	1	0	0	1	2	0	0	0	1	0	1	0	0	0	2	0	0	0
Lallemantia_royleana	0	0	0	1	2	2	0	2	0	0	0	0	0	0	0	0	0	0
Lappula_sinaica	0	0	0	0	0	0	0	0	0	0	0	2	0	0	0	0	0	0
Lepidium_draba	0	0	0	2	0	0	0	0	0	0	0	0	0	0	0	0	0	0
Lepyrodiclis_sp_	0	0	0	0	0	0	0	0	0	0	0	0	2	0	0	0	0	0
Marrubium_anisodon	0	0	2	0	0	0	0	0	0	0	0	0	0	2	0	0	0	0
Medicago_lupulina	0	0	0	0	0	0	0	0	1	0	0	0	0	0	0	0	0	0
Medicago_sativa	0	0	0	0	0	0	0	0	0	0	0	0	0	0	0	0	0	0
Nepeta_saccharata	0	0	0	0	0	0	0	0	0	0	0	2	0	0	0	0	0	0
Onobrychis_cornuta	0	0	0	0	0	0	0	0	0	0	1	0	0	0	0	0	0	0
Papaver_dubium	0	0	0	1	0	2	0	0	0	0	0	0	0	0	0	0	0	0
Parietaria_judaica	0	0	0	0	0	0	0	0	0	0	0	0	0	1	0	0	0	0
Poa_bulbosa	20	8	0	20	7	0	6	0	0	6	0	3	0	0	0	10	0	0
Polygonum_polycnemoides	0	0	0	0	0	0	0	0	0	0	0	0	0	0	0	0	0	0
Rosa_pumila	0	0	0	0	0	0	0	0	0	0	0	0	1	0	0	0	0	0
Sanguisorba_minor	0	0	0	0	0	0	0	0	0	0	0	0	0	1	0	0	0	0
Scandix_stellata	0	0	0	0	0	0	0	0	0	0	0	0	0	0	2	0	0	0
Scorzonera_raddeana	0	0	0	0	0	0	0	0	0	0	0	0	2	0	0	0	0	0
Scrophularia_scoparia	0	0	0	0	0	0	0	0	0	0	0	0	0	0	0	0	0	0
Silene_swertiaefolia	0	1	0	0	0	0	0	0	0	0	0	1	0	0	0	1	0	0
Stellaria_alsinoides	0	0	0	0	0	0	0	0	0	0	0	0	3	0	0	0	0	0
Stipa_hohenackeriana	0	0	0	0	0	0	0	0	0	0	0	0	0	0	1	0	0	0
Taeniatherum_caputmedusae	0	0	4	0	0	0	0	15	0	0	0	0	0	10	0	0	0	0
Tanacetum_khorassanicum	0	0	0	0	0	0	0	0	0	0	0	0	0	0	0	0	0	2
Tragopogon_collinus	0	0	0	0	0	0	0	0	0	0	0	0	0	0	0	0	0	0
Veronica_sp_	0	0	0	0	0	0	0	0	0	0	0	0	0	0	0	0	0	0
Verbascum_songaricum	0	1	0	0	0	0	0	0	0	0	0	0	0	0	0	0	0	0
Verbascum_sp_	0	0	1	0	0	1	0	0	1	0	0	0	0	0	0	0	0	0
Ziziphora_clinopodioides	0	0	0	0	0	0	0	0	0	0	0	0	0	0	0	0	0	4
Ziziphora_tenuior	0	0	0	0	0	0	0	0	0	0	0	0	0	0	0	0	0	5

Seseli transcaucasicum Arabchah

CFS

Acantholimon_bodeanum	0	0	0	0	25	0	25	0	0	60	0	0	0	0	0	0	0	0	0
Acanthophyllum_glandulosum	25	20	0	7	0	0	0	0	0	0	0	0	0	0	0	0	0	0	0
Allium_monophyllum	0	0	0	0	0	0	0	0	5	0	0	0	0	0	0	0	0	0	0
Allium_xiphopetalum	0	0	0	0	0	0	0	0	0	0	0	0	0	0	0	0	0	0	0
Alyssum_desertorum	0	0	0	0	0	5	0	0	0	0	0	0	0	0	5	0	0	0	0
Alyssum_hezarmasjedensis	0	0	0	0	0	0	0	0	0	0	0	0	0	0	0	0	0	0	0
Asperula_glomerata	0	0	0	0	0	0	0	0	0	0	0	0	0	0	0	0	0	0	20
Astragalus_kochak	10	0	0	0	0	0	0	0	0	5	0	0	0	0	5	0	0	0	0
Astragalus_radeii	0	0	0	0	0	0	0	0	0	0	0	0	0	0	0	0	0	0	0
Astragalus_red	0	0	0	0	0	0	0	0	0	0	0	0	0	0	0	0	0	0	0
Astragalus_white	0	0	0	0	0	0	0	0	0	0	0	0	0	0	0	30	0	0	0
Carex_stenophylla	16	0	0	0	0	0	0	0	0	0	0	10	0	0	0	0	0	0	0
Ceratocephala_testiculata	0	0	0	0	0	0	0	0	0	0	0	0	0	0	0	0	0	0	0
Chaerophyllum_khorossanicum	0	0	0	0	0	0	0	0	0	0	0	0	0	0	0	0	0	0	0
Cirsium_strigosum	10	10	32	10	0	0	0	0	0	0	0	0	0	0	0	0	25	0	0
Colchicum_robustum	0	0	0	0	0	0	0	0	0	0	0	0	0	0	5	0	0	0	0
Cousinia_microcarpa	0	0	0	0	0	0	0	0	0	0	0	0	0	0	0	0	0	0	0
Cousinia_multiloba	0	0	30	10	0	10	0	0	0	0	0	0	0	0	0	0	0	0	0
Crepis_multicaulis	0	0	0	0	0	0	0	0	0	0	0	0	0	0	0	0	0	0	0
Elwendia_afghanica	0	0	0	0	0	0	0	0	0	0	0	0	0	0	0	0	0	0	0
Euphorbia_aucheri	0	0	0	5	0	0	0	0	0	0	0	0	0	0	0	0	0	0	0
Euphorbia_boissieriana	0	15	0	0	0	20	0	0	10	0	0	0	0	0	0	15	15	0	0
Euphorbia_microsciadia	15	0	0	0	0	0	0	0	0	0	0	0	0	0	0	0	0	15	0
Fessia_khorassanica	0	0	0	0	0	0	0	0	0	0	0	0	0	0	3	0	0	0	0
Festuca_sp	0	0	0	0	0	0	0	0	0	0	0	0	0	0	0	0	0	0	0
Gagea_reticulata	0	0	0	0	0	0	0	0	0	0	0	0	0	0	0	0	0	0	0
Galium_spurium	0	0	0	0	0	0	0	0	0	0	0	0	0	0	0	0	0	0	0
Gallium_humifusum	0	0	0	0	0	0	0	0	0	0	0	0	0	0	5	0	15	0	0
Holosteum_glutinosum	0	0	0	0	0	0	0	0	0	0	0	0	0	0	0	0	0	3	0
Hyoscyamus_senecionis	0	0	0	0	0	0	0	0	0	0	0	0	0	0	0	0	0	0	15
Iris_loczyi	0	0	0	0	0	0	0	0	0	0	0	0	0	0	0	0	0	0	0
Lappula_microcarpa	0	5	0	0	10	0	0	0	0	0	0	0	0	0	0	0	0	0	0
Onobrychis_cornuta	0	20	0	0	25	0	40	90	0	0	20	0	45	15	20	10	40	50	0
Poa_bulbosa	0	0	0	0	20	10	5	0	14	25	0	0	20	15	0	0	0	9	0
Poa_sp	0	0	0	0	0	0	0	0	0	0	0	0	0	0	0	0	0	0	0
Polygonum_afghanicum	0	0	0	0	0	0	10	0	0	0	0	0	0	0	0	0	0	0	0
Polygonum_arenastrum	0	0	0	0	0	0	0	0	5	0	0	5	0	0	0	0	0	0	0
Rannunculus_afghanicus	0	0	0	0	0	0	0	0	0	0	0	0	6	0	0	0	15	0	0
Silene_odontopetala	0	0	0	0	0	0	0	0	0	5	0	0	0	0	0	0	0	0	0
Silene_red	0	0	0	0	0	0	0	0	0	0	0	0	0	0	0	0	0	0	0
Stachys_lavandulifolia	0	0	0	12	0	0	0	0	0	0	0	0	0	0	0	0	0	0	0
Stellaria_alsinoides	0	0	0	0	0	0	0	0	0	0	0	0	0	0	0	0	0	0	0
Stipa_arabica	0	0	0	0	0	0	0	0	0	0	0	0	0	0	0	0	0	0	0
Tanacetum_turcomanicum	0	0	0	0	0	0	12	0	0	0	0	0	0	0	0	0	0	0	25
Taraxacum_brevirostre	0	0	3	0	8	10	0	0	16	9	0	15	15	0	0	6	0	9	0
Taraxacum_iranicum	0	0	0	0	0	0	0	0	0	0	0	0	0	0	0	0	0	0	0
Taraxacum_serotinum	0	0	0	0	0	0	0	0	0	0	0	0	0	0	0	0	0	0	0
Taraxacum_sp	0	0	0	6	0	0	0	0	0	0	0	0	0	0	0	0	0	0	0
Thymus_transcaspicus	0	0	15	0	15	0	15	0	10	0	0	0	0	20	15	15	0	15	0
Valeriana_sp	0	0	0	0	0	0	0	0	0	0	0	0	0	0	10	0	0	0	0
Veronica_beccabonga	0	0	0	0	0	0	0	0	0	0	0	0	0	0	0	20	0	0	0
Veronica_biloba	3	0	3	3	0	0	0	0	0	0	3	0	5	0	0	0	0	0	0
Veronica_kopetdaghensis	0	0	0	0	0	0	0	0	0	0	0	0	0	0	0	0	5	0	0
Ziziphora_clinopodiodes	0	0	0	0	0	25	0	0	0	0	10	5	0	0	0	0	0	0	0

WFS

Acantholimon_bodeanum	0	0	0	10	0	0	0	0	0	20	30	0	10	40	0	0	18	0	0
Acanthophyllum_glandulosum	0	0	0	15	0	0	0	0	0	0	0	0	0	0	0	0	0	0	0
Allium_monophyllum	0	0	0	0	0	0	20	9	0	0	0	0	0	0	0	12	6	0	0
Allium_xiphopetalum	0	0	0	0	0	0	0	0	0	0	0	0	5	0	0	0	6	0	0
Alyssum_desertorum	0	0	0	0	0	0	6	3	15	0	0	0	0	9	0	0	3	0	0
Alyssum_hezarmasjedensis	0	0	0	0	0	0	0	0	0	0	0	0	18	0	0	0	0	0	0
Asperula_glomerata	0	0	0	0	0	0	10	0	3	5	0	0	8	0	5	0	0	0	0
Astragalus_kochak	0	0	0	0	0	0	0	0	0	0	0	0	0	0	0	0	0	0	0
Astragalus_radeii	5	5	0	0	0	5	0	0	0	0	0	0	0	0	0	4	0	0	0
Astragalus_red	0	0	0	0	0	0	0	0	0	0	0	0	0	5	0	0	0	0	5
Astragalus_white	0	0	0	0	0	0	0	0	0	0	0	0	0	0	0	0	0	0	0
Carex_stenophylla	16	25	0	0	0	0	0	0	3	0	4	3	0	0	0	0	0	0	0
Ceratocephala_testiculata	0	0	0	0	0	0	0	0	0	0	0	0	0	0	0	0	0	2	5
Chaerophyllum_khorossanicum	0	0	0	0	0	0	10	8	0	0	0	0	0	0	0	0	0	0	0
Cirsium_strigosum	5	10	0	0	0	0	0	0	0	0	0	0	0	0	0	0	0	0	0
Colchicum_robustum	0	0	0	0	0	0	0	0	5	0	0	0	0	20	12	0	0	0	0
Cousinia_microcarpa	0	0	10	0	0	20	5	0	10	10	15	0	0	0	9	0	0	0	0
Cousinia_multiloba	0	0	0	0	20	0	0	0	15	0	0	30	0	0	0	0	0	0	0
Crepis_multicaulis	0	0	0	0	0	0	0	0	0	5	0	0	0	0	0	0	0	0	0
Elwendia_afghanica	18	10	10	0	24	30	0	0	0	0	6	0	5	0	0	0	10	0	0
Euphorbia_aucheri	5	0	5	0	0	0	2	5	0	0	0	5	0	0	0	0	0	0	0
Euphorbia_boissieriana	25	0	0	0	15	10	0	0	0	0	0	0	0	0	0	0	0	0	0
Euphorbia_microsciadia	0	15	0	0	0	0	0	0	0	0	0	0	0	10	0	0	0	0	0
Fessia_khorassanica	0	0	0	0	0	0	3	0	0	0	0	2	0	0	0	0	0	0	0
Festuca_sp	0	0	0	0	0	0	0	0	0	5	0	0	10	5	0	0	0	0	0
Gagea_reticulata	0	3	0	3	0	0	0	0	0	0	3	0	0	0	0	0	0	0	0
Galium_spurium	0	0	0	15	0	0	0	0	0	0	0	0	0	0	0	0	0	0	0
Gallium_humifusum	0	0	0	0	0	0	0	0	0	0	0	0	0	0	0	0	0	0	0
Holosteum_glutinosum	0	0	0	0	0	0	0	0	0	0	0	0	0	0	0	0	0	0	0
Hyoscyamus_senecionis	0	0	0	0	0	0	0	0	0	0	0	0	0	0	0	0	0	0	10
Iris_loczyi	15	50	20	20	0	0	0	0	0	0	25	40	0	0	0	0	0	0	0
Lappula_microcarpa	0	0	0	0	0	0	0	0	0	0	0	0	0	0	0	0	3	0	0
Onobrychis_cornuta	30	0	35	0	25	10	35	0	0	0	10	0	45	0	0	0	0	0	0
Poa_bulbosa	0	0	5	0	0	0	6	0	9	0	25	0	10	9	15	25	15	5	3
Poa_sp	0	0	0	0	0	0	0	0	0	0	3	0	0	0	0	0	0	0	0
Polygonum_afghanicum	0	0	0	0	0	0	0	0	10	0	0	0	0	0	0	0	0	0	0
Polygonum_arenastrum	0	0	5	5	0	0	0	0	0	0	0	0	0	0	0	0	0	3	0
Rannunculus_afghanicus	0	0	0	0	0	0	0	0	0	0	0	0	0	0	0	0	0	0	0
Silene_odontopetala	0	0	0	0	0	0	0	0	0	0	0	0	0	0	0	0	0	0	0
Silene_red	0	0	0	0	0	0	0	10	0	0	0	0	0	0	0	0	0	0	0
Stachys_lavandulifolia	0	0	0	0	0	0	0	0	0	0	0	0	0	0	0	0	0	0	0
Stellaria_alsinoides	0	0	0	0	0	0	0	0	0	0	0	0	0	0	5	0	0	0	0
Stipa_arabica	0	0	0	0	0	0	0	0	0	5	0	0	0	0	0	0	0	0	0
Tanacetum_turcomanicum	0	0	0	0	0	0	0	0	0	0	0	0	0	0	10	35	15	0	15
Taraxacum_brevirostre	0	0	0	0	0	0	0	15	0	0	0	12	8	0	0	15	6	12	0
Taraxacum_iranicum	0	0	0	0	0	0	0	0	0	0	0	0	0	0	15	0	12	0	0
Taraxacum_serotinum	0	0	0	0	0	0	10	0	0	0	0	0	0	0	0	0	0	0	0
Taraxacum_sp	0	0	0	0	0	0	0	0	0	0	0	0	0	0	0	0	0	0	0
Thymus_transcaspicus	0	0	0	15	0	0	15	15	0	30	25	0	15	0	10	0	20	0	0
Valeriana_sp	0	0	0	0	0	0	0	0	0	0	0	0	0	0	0	0	0	0	0
Veronica_beccabonga	0	0	0	0	0	0	0	0	0	0	0	0	0	0	0	0	0	0	0
Veronica_biloba	4	10	10	11	0	0	0	0	0	5	0	8	0	0	4	4	0	15	10
Veronica_kopetdaghensis	0	0	0	0	0	2	0	0	0	0	0	0	0	0	0	0	0	0	0
Ziziphora_clinopodiodes	0	0	0	0	0	0	10	0	0	0	0	0	15	0	0	0	10	0	0
